# Series Representations for Uncertain Fractional IVPs in the Fuzzy Conformable Fractional Sense

**DOI:** 10.3390/e23121646

**Published:** 2021-12-07

**Authors:** Malik Bataineh, Mohammad Alaroud, Shrideh Al-Omari, Praveen Agarwal

**Affiliations:** 1 Department of Mathematics, Jordan University of Science and Technology, Irbid 22110, Jordan; msbataineh@just.edu.jo; 2 Department of Mathematics, Faculty of Arts and Science, Amman Arab University, Amman 11953, Jordan; m.alaroud@aau.edu.jo; 3 Department of Physics and Basic Sciences, Faculty of Engineering Technology, Al-Balqa Applied University, Amman 11134, Jordan; 4 Department of Mathematics, Anand International College of Engineering, Jaipur 302012, India; goyal.praveen2011@gmail.com

**Keywords:** triangular fuzzy number, residual power series method, fractional calculus, approximate solution

## Abstract

Fuzzy differential equations provide a crucial tool for modeling numerous phenomena and uncertainties that potentially arise in various applications across physics, applied sciences and engineering. Reliable and effective analytical methods are necessary to obtain the required solutions, as it is very difficult to obtain accurate solutions for certain fuzzy differential equations. In this paper, certain fuzzy approximate solutions are constructed and analyzed by means of a residual power series (RPS) technique involving some class of fuzzy fractional differential equations. The considered methodology for finding the fuzzy solutions relies on converting the target equations into two fractional crisp systems in terms of ρ-cut representations. The residual power series therefore gives solutions for the converted systems by combining fractional residual functions and fractional Taylor expansions to obtain values of the coefficients of the fractional power series. To validate the efficiency and the applicability of our proposed approach we derive solutions of the fuzzy fractional initial value problem by testing two attractive applications. The compatibility of the behavior of the solutions is determined via some graphical and numerical analysis of the proposed results. Moreover, the comparative results point out that the proposed method is more accurate compared to the other existing methods. Finally, the results attained in this article emphasize that the residual power series technique is easy, efficient, and fast for predicting solutions of the uncertain models arising in real physical phenomena.

## 1. Introduction

Fuzzy set theory is of considerable interest in mathematics that generalizes the classical probability. The theory fulfills the need to express information of human knowledge in mathematical forms. Since its inception [1], it has been successfully applied in many fields, most notably in the areas of decision making, modeling uncertainty, pattern recognition, image processing, machine learning, economics, and artificial intelligence [2,3]. In the last few years, modeling uncertainty has gained the attention of numerous scholars as one of the most popular theories of describing physical phenomena using fuzzy fractional initial value problems (IVPs). In some cases, simulation and modeling of a real physical phenomenon shows information about issues associated with uncertainty. Such uncertainty may result from several factors, including the process of data collection and measurement errors, determining the initial data, and so forth. Therefore, it is necessary to develop convenient and reliable methods to clarify the presence of uncertainty in parameters, variables, and constants in a mathematical structure of different phenomena that can appropriately address the fuzzy fractional IVPs and study their qualitative and quantitative physical behavior.

Fuzzy differentiation and integration in recent years has witnessed fast-growing application in diverse and widespread fields in natural science and engineering, for instance, electrical engineering, synchronized hyperchaotic systems, quantum optics, chaotic systems, medicine, and many others (see [4,5,6,7,8]). In the literature, different fractional derivative operators have been proposed and improved, such as the Riemann–Liouville, Caputo, Caputo–Fabrizio, and conformable concepts (see [9,10,11,12]). Consequently, various numerical methods have been developed to deal with these fractional operators; for further applications, refer to [13,14,15,16,17,18]. The investigation of FFDEs and their solutions was initially established by Agarwal et al. in [19], in which they solved FFDE with respect to Riemann–Liouville differentiability. This contribution has spurred numerous researchers to devote their interest towards the study of the theoretical results of the existence and uniqueness of solutions side by side with the numerical approximation methods of FFDEs, including the reproducing kernel Hilbert space method, the fractional Euler method, the fuzzy Laplace transform method, the variational iteration method, the Adomian decomposition method, the Jacobi operational matrix method, the Taylor series expansion method, and others (see [20,21,22,23]).

The basic purpose of this analysis is to develop a framework to investigate the fuzzy approximate solutions of a certain class of fuzzy fractional IVP with respect to fuzzy conformable fractional derivative by applying the residual power series (RPS) technique. The proposed technique was initially introduced as an attractive novel numeric-analytic approach for constructing the series solutions for fuzzy IVPs by determining the component values of the expansion series. It depends on the fractional derivative of the so-called truncation residual error function in each stage of finding the solution. RPS has been widely used to find out the solutions of linear and nonlinear issues of fractional differential and fractional integrodifferential equations, including fractional Newell–Whitehead–Segel equation [24], fractional Sawada–Kotera–Ito, Lax, and Kaup–Kupershmidt equations [25], time-fractional Fokker–Planck equations [26], fractional Kundu–Eckhaus and massive Thirring models [27], coupled fractional resonant Schrödinger equation [28], and the fractional Sharma–Tasso–Olever equation [29]. The proposed algorithm is straightforward, accurate and powerful for creating a series of solutions for different models that occur in applied mathematics without terms of perturbation, discretization, and linearization. For more information about advanced different and approximate methods, refer to [30,31,32,33,34,35] and references therein.

In this analysis, we intend to design an efficient algorithm capable of implementing a direct and accurate iterative method to find approximate solutions to the fuzzy system in view of the conformable fractional sense of the domain of interest. The rest of this analysis is organized as follows. In the next section, some mathematical preliminaries and basic definitions related to fuzzy numbers, fuzzy conformable differentiation and fractional Taylor’s formula are reviewed. In Section 3, the formulation of fuzzy fractional IVPs of order β is presented. The principle of the RPS method to detect the solutions of fuzzy fractional IVPs is introduced in Section 4. In Section 5, two linear FFDEs with appropriate fuzzy initial data under fuzzy conformable differentiability are tested to illustrate the simplicity and potential of the RPS approach for determining the approximate solutions. Finally, the conclusion of this work is given in Section 6.

## 2. Preliminaries

This section provides the fundamental definitions and preliminary results for elucidating sufficient fuzzy analysis theory, to enable us to investigate the fuzzy approximated solutions for certain classes of FFDEs. Throughout this article, ${ℜ}_{F}$ refers to the set of all fuzzy numbers.

**Definition** **1.** *[36] The β-th conformable fractional derivative starting from η of a function $\varphi :\left[\eta ,\infty \right)\to \mathbb{R}$ is denoted $C^{\beta }$ and defined as:*

$$C^{\beta }\varphi \left(t\right)=\underset{\varepsilon \to 0}{\lim}\frac{\varphi^{\left(m-1\right)}\left(t+\varepsilon {\left(t-\eta \right)}^{m-\beta }\right)-\varphi^{\left(m-1\right)}\left(t\right)}{\varepsilon }\;,\;\beta \in \left(m-1,m\right]\;,\;t>\eta ,$$

*and $C^{\beta }\varphi \left(\eta \right)=\underset{t\to \eta^{+}}{\lim}C^{\beta }\varphi \left(t\right)$ provided that $\underset{t\to \eta^{+}}{\lim}C^{\beta }ⱳ\left(t\right)$ exists and $\varphi \left(t\right)$ is $\left(m-1\right)$-differintiable in some $\left(0,\eta \right),$ η>0.*

**Definition** **2.** *[5] A fuzzy number is defined as a fuzzy set $\omega :ℜ\to \left[0,1\right]$ such that*

- *ω is upper semi-continuous, i.e., $\lim_{t\to \xi }\omega \left(t\right)\geq \omega \left(\xi \right)$, ⩝ξ∈ℜ.*
- *ω is convex, i.e., for each ξ,η∈ℜ, and 0≤γ≤1, we have $\omega \left(\gamma \xi +\left(1-\gamma \right)\eta \right)\geq \min\left(\omega \left(\xi \right),\;\omega \left(\eta \right)\right)$.*
-
ω *is normal, i.e., there is at least one point* ξ∈ℜ *such that* $\omega \left(t\right)=1.$

-
${\left[\omega \right]}_{0}=\bar{\left\{\xi \in ℜ:\omega \left(\xi \right)>0\right\}}$ *is compact set.*

**Theorem** **1.** *[6]* *Let* $\underline{\omega },\bar{\omega }:\left[0,1\right]\to ℜ$ *satisfy the following conditions:*

*(i)* 
$\underline{\omega }$ *is a bounded non-decreasing function.*
*(ii)* 
$\bar{\omega }$ *is a bounded non-increasing function.*
*(iii)*  $\underline{\omega }\left(1\right)\leq \bar{\omega }\left(1\right)$.
*(iv)*  *For each* $i\in \left(0,1\right]$ *,* $\lim_{\rho \to i^{-}}\underline{\omega }\left(\rho \right)=\underline{\omega }\left(i\right)$ *and* $\lim_{\rho \to i^{-}}\bar{\omega }\left(\rho \right)=\bar{\omega }\left(i\right)$ *.*
*(v)* 
$\lim_{\rho \to 0^{+}}\underline{\omega }\left(\rho \right)=\underline{\omega }\left(0\right)$ *and* $\lim_{\rho \to 0^{+}}\bar{\omega }\left(\rho \right)=\bar{\omega }\left(0\right)$ *.*

*Then,* $\omega :\left[0,1\right]\to ℜ$ *given by* $\omega \left(t\right)=\sup\left\{\rho |\underline{\omega }\left(\rho \right)\leq t\leq \bar{\omega }\left(\rho \right)\right\}$ *is a fuzzy number with parameterization* $\left[{\underline{\omega }}_{\rho },{\bar{\omega }}_{\rho }\right]$ *. Furthermore, if* $\omega :\left[0,1\right]\to ℜ$ *is a fuzzy number with parameterization* $\left[{\underline{\omega }}_{\rho },{\bar{\omega }}_{\rho }\right]$ *, then the functions* ${\underline{\omega }}_{\rho }$ *and* ${\bar{\omega }}_{\rho }$ *satisfy the aforesaid conditions (i)–(v). Consequently, the arbitrary fuzzy number* ω *can be presented as an ordered pair of functions* $\left({\underline{\omega }}_{\rho },{\bar{\omega }}_{\rho }\right).$

**Definition** **3.** *[7] For $D:\;{ℜ}_{F}\times {ℜ}_{F}\to {ℜ}^{+}\cup \left\{0\right\},$ the mapping $D\left(\omega ,\varphi \right)$ can be defined as $D\left(\omega ,\varphi \right)=\sup_{0\;\leq \rho \leq 1}D_{H}\left\{{\left[\omega \right]}_{\rho },{\left[\varphi \right]}_{\rho }\right\}$ for arbitrary fuzzy numbers ω=($\underline{\omega },\;\bar{\omega })$ and $\varphi =\left(\underline{\varphi },\;\bar{\varphi }\right)$, where $D_{H}$ is the Hausdorff metric: $D_{H}\left\{{\left[\omega \right]}_{\rho },{\left[\varphi \right]}_{\rho }\right\}=\max\left\{\left|{\underline{\omega }}_{\rho }-{\underline{\varphi }}_{\rho }\right|,\left|{\bar{\omega }}_{\rho }-{\bar{\varphi }}_{\rho }\right|\right\}$.*

**Definition** **4.** *[7] The β-th fuzzy conformable fractional derivative for fuzzy function $\omega :\left(a,b\right)\to {ℜ}_{F}$ for β>0 is denoted by $C^{\beta }$ and defined by*

$$\left(C^{\beta }\omega \right)\left(t\right)=\underset{\zeta \to 0^{+}}{\lim}\frac{\omega \left(t+\zeta t^{1-\beta }\right)⊖\omega \left(t\right)}{\zeta }=\underset{\zeta \to 0^{+}}{\lim}\frac{\omega \left(t\right)⊖\omega \left(t-\zeta t^{1-\beta }\right)}{\zeta },\;\beta \in \left(0,1\right].$$

**Remark** **1.** *We define $C^{\beta }\omega \left(0\right)=\underset{t\to 0^{+}}{\lim}C^{\beta }\omega \left(t\right)$ provided the limit is exists. Furthermore, ω is β-th fuzzy conformable differentiable whenever $C^{\beta }\omega \left(t\right)$ exists for β>0.*

**Definition** **5.** *[7] For $t_{0}\in \left[a,b\right]$, a>0, and β>0, we say that $\omega :\left[a,b\right]\to {ℜ}_{F}$ is strongly generalized βth-fuzzy conformable differentiable at $t_{0}$ if there exists an element $C^{\beta }\omega \left(\tau \right)\in {ℜ}_{F}$ such that either:*

*(i)*  *The H-differences* $\omega \left(t_{0}+\zeta t_{0}^{1-\beta }\right)⊖\omega \left(t_{0}\right)$ *,* $\omega \left(t_{0}\right)⊖\omega \left(t_{0}-\zeta t_{0}^{1-\beta }\right)$ *exist for each sufficiently small* ζ>0 *, and* $\underset{\zeta \to 0^{+}}{\lim}\frac{\omega \left(t_{0}+\zeta t_{0}^{1-\beta }\right)⊖\omega \left(t_{0}\right)}{\zeta }=\underset{\zeta \to 0^{+}}{\lim}\frac{\omega \left(t_{0}\right)⊖\omega \left(t_{0}-\zeta t_{0}^{1-\beta }\right)}{\zeta }=C^{\beta }\omega \left(t_{0}\right)$ *.*
*(ii)*  *The H-differences*$\omega \left(t_{0}\right)⊖\omega \left(t_{0}+\zeta t_{0}^{1-\beta }\right)$*,*$\omega \left(t_{0}-\zeta t_{0}^{1-\beta }\right)⊖\omega \left(t_{0}\right)$*exist, for each sufficiently small*ζ>0*, and*$\underset{\zeta \to 0^{+}}{\lim}\frac{\;\omega \left(t_{0}\right)⊖\omega \left(t_{0}+\zeta t_{0}^{1-\beta }\right)}{-\zeta }=\underset{\zeta \to 0^{+}}{\lim}\frac{\omega \left(t_{0}-\zeta t_{0}^{1-\beta }\right)⊖\omega \left(t_{0}\right)}{-\zeta }=C^{\beta }\omega \left(t_{0}\right)$.

*It is worth mentioning here that the limits are taken in the metric space $\left({ℜ}_{F},D\right)$.*

**Remark** **2.** *If ω  is fuzzy differentiable for any point $t\in \left(a,b\right)$ in terms of (i) of Definition 2.5, then ω is a $\left(1;\beta \right)$-fuzzy conformable differentiable on $\left[a,b\right]$ and its derivative is $C_{1}^{\beta }\omega \left(t\right)$. Likewise, ω is a $\left(2;\beta \right)$-fuzzy conformable differentiable on $\left[a,b\right]$, if ω is fuzzy differentiable for any point $t\in \left(a,b\right)$ in terms of (ii) of Definition 2.5 and its derivative is $C_{2}^{\beta }\omega \left(t\right)$.*

**Theorem** **2.** *[7]* *Assume that* $\omega :\left[a,b\right]\to {ℜ}_{F}$ *is a fuzzy function satisfies the following conditions:*

*(i)*  *For each $t\in \left[a,b\right],$ there exists δ>0 such that the H-differences: $\omega \left(t+\zeta t^{1-\beta }\right)⊖\omega \left(t\right)$ and $\omega \left(t\right)⊖\omega \left(t-\zeta t^{1-\beta }\right)$ exists for all* *ζ* *∈ [0,* *δ* *).*
*(ii)*  *For each* $t\in \left[a,b\right]$ *and* h>0 *there exists a constant* ℓ>0 *such that*

$D_{H}\left(\frac{\;\omega \left(t+\zeta t^{1-\beta }\right)-\omega \left(t\right)}{\zeta ,\;C^{\beta }\omega \left(t\right)}\right)<h,$ *and* $D_{H}\left(\frac{\;\omega \left(t\right)-\omega \left(t-\zeta t^{1-\beta }\right)}{\zeta ,\;C^{\beta }\omega \left(t\right)}\right)<h,$

*for all* $\zeta \in \left[0,\ell \right)$ *. Then, the set of functions* ${\left[\omega \left(t\right)\right]}_{\rho }$ *is* β *-th conformable differentiable and its derivative is* ${\left[C^{\beta }\omega \left(t\right)\right]}_{\rho }=\left[C^{\beta }{\underline{\omega }}_{\rho }\left(t\right),C^{\beta }{\bar{\omega }}_{\rho }\left(t\right)\right]$ *, where* ${\left[\omega \left(t\right)\right]}_{\rho }=\left[{\underline{\omega }}_{\rho }\left(t\right),{\bar{\omega }}_{\rho }\left(t\right)\right]$ *for each* $\rho \in \left[0,1\right].$

*Next, theorems assist us to convert the FFDEs into a system of ordinary fractional differential equations.*

**Theorem** **3.** *[7] Assume that* $\omega :\left[a,b\right]\to {ℜ}_{F}$ *is a fuzzy function. Let* ${\left[\omega \left(t\right)\right]}_{\rho }=\left[{\underline{\omega }}_{\rho }\left(t\right),{\bar{\omega }}_{\rho }\left(t\right)\right]$ *for each* $\rho \in \left[0,1\right]$ *. Then,*

(i) *If ω is $\left(1;\beta \right)$-fuzzy conformable differentiable, then ${\underline{\omega }}_{\rho }$ and ${\bar{\omega }}_{\rho }$ are β-th conformable differentiable functions on $\left[a,b\right]$ and ${\left[C^{\beta }\omega \left(t\right)\right]}_{\rho }=\left[C^{\beta }{\underline{\omega }}_{\rho }\left(t\right),C^{\beta }{\bar{\omega }}_{\rho }\left(t\right)\right]$.*
(ii) *If ω is $\left(2;\beta \right)$-fuzzy conformable differentiable, then ${\underline{\omega }}_{\rho }$ and ${\bar{\omega }}_{\rho }$ are β-th conformable differentiable functions on $\left[a,b\right]$ and ${\left[C^{\beta }\omega \left(t\right)\right]}_{\rho }=\left[C^{\beta }{\bar{\omega }}_{\rho }\left(t\right),\;C^{\beta }{\underline{\omega }}_{\rho }\left(t\right)\right]$.*

**Definition** **6.** *[37] A fractional expansion representation at t=η has the following form:*

$$\overset{\infty }{\underset{k=0}{\sum }}a_{k}\left(t-\eta \right){\;}^{\beta k}=a_{0}+a_{1}{\left(t-\eta \right)}^{\beta }+a_{2}{\left(t-\eta \right)}^{2\beta }+\cdots ,$$

*where* 0≤n−1<β≤n *, and* t≥η *is a fractional power series (PS) about* η *.*

**Theorem** **4.** *[38] Suppose that*$\varphi \left(t\right)$*has the following fractional PS representation at*t=η:

$$\varphi \left(t\right)=\overset{\infty }{\underset{k=0}{\sum }}a_{k}\left(t-\eta \right){\;}^{\beta k},0\leq n-1<\beta \leq n,t\in \left[\;\eta ,\eta +R\right),$$

*where*$\varphi \left(t\right)\in C\left[\eta ,\eta +R\right)$*, then the unknown functions*$a_{k}$*are in the form*$a_{k}=\frac{C^{k\beta }\varphi \left(\eta \right)}{\beta^{k}k!}$*for*k=0,1,2,…, such that $C^{k\beta }=C^{\beta }\cdot C^{\beta }\cdots C^{\beta },$*k-times.*

**Remark** **3.** It should be mentioned that there is an exciting recent work on the conformable Euler method for finite difference discretization of FIVPs [39,40] showing that the fractional Taylor expansions in terms of the conformable fractional derivative presented in [36] is valid for β=1. An alternative definition of the conformable fractional derivative introduced in [40] based on the exact spectral derivative discretization finite difference method showing that the conformable fractional derivative [36] is a fractional change of a variable rather that a fractional operator. In view of the results of [*], Definition 6 and Theorem 4 are incorrect, and the RPS results-based thereon can therefore be improved.

**Definition** **7.** *[40] Given a real-valued function on $\left[0,\;\infty \right)$, the conformable fractional derivative has the following alternative definition:*

$$T_{t}^{\beta }\varphi \left(t\right)={\;}_{0}^{C}T_{t}^{\beta }\varphi \left(t\right)\equiv \underset{h\to 0}{\lim}{\;}_{0}^{CFD}\Delta_{t}^{\beta }\varphi \left(t\right)=\beta \underset{h\to 0}{\lim}\frac{\varphi \left(t+h\right)-\varphi \left(t\right)}{{\left(t+h\right)}^{\beta }-t^{\beta }},$$

*where* ${\;}_{0}^{C}T_{t}^{\beta }\varphi \left(0\right)$ *is understood to mean* ${\;}_{0}^{C}T_{t}^{\beta }\varphi \left(0\right)=\underset{t\to 0^{+}}{\lim}{}_{0}^{C}T_{t}^{\beta }\varphi \left(t\right)$ *.*

## 3. Fuzzy Conformable Fractional Initial Value Problems

Recently, fuzzy DEs have emerged as a powerful instrument for mathematical modeling of numerous real-life phenomena. In this section, let us consider the following fuzzy fractional IVPs of order β:

(1) $$C^{\beta }\omega \left(t\right)=F\left(t,\omega \left(t\right)\right),\;a\leq \;t\leq b,\;\beta \in \left(\left.0,1\right]\right.,$$

with the fuzzy initial condition

(2) $$\omega \left(a\right)=\sigma$$

where $C^{\beta }$ indicates the fuzzy conformable fractional derivative of order β, $F:\left[a,b\right]\times {ℜ}_{F}\to {ℜ}_{F}$ is a continuous fuzzy-valued function, $\sigma \in {ℜ}_{F}$ and $\omega \left(t\right)$ is unknown analytical function to be determined. Consequently, if $F\left(\cdot \right)$ is a crisp function, then the solution $\omega \left(t\right)$ of IVPs (1) and (2) is a crisp. Otherwise, if $F\left(\cdot \right)$ is a fuzzy function, then the IVPs (1) and (2) may possess only fuzzy solution $\omega \left(t\right)$. Anyhow, we assume that $F\left(\cdot \right)$ is a fuzzy function.

The $\left(m\right)$-fuzzy solution of the fuzzy fractional IVPs (1) and (2) is a function $\omega :\left[a,b\right]\to \;{ℜ}_{F}$ which is (m;β)-fuzzy conformable differentiable and satisfies (1) and (2). To obtain the fuzzy solution $\omega \left(t\right),$ we firstly convert the fuzzy fractional IVPs (1) and (2) into equivalent systems of fractional IVPs, based upon the type of the fuzzy conformable differentiability and the fuzzy solution ω which satisfies the above conditions of Theorem 2. Then, by rewriting $C^{\beta }\omega \left(t\right),$ $\omega \left(t\right),$ and the initial data $\omega \left(a\right)$, respectively, as a ρ-cut representation: $\left[C^{\beta }{\underline{\omega }}_{\rho }\left(t\right),C^{\beta }{\bar{\omega }}_{\rho }\left(t\right)\right]$,$\left[{\underline{\omega }}_{\rho }\left(t\right),{\bar{\omega }}_{\rho }\right.\left(t\right)]$, and $\left[{\underline{\omega }}_{\rho }\left(a\right),{\bar{\omega }}_{\rho }\right.\left(a\right)]=\left[{\underline{\delta }}_{\rho },{\bar{\delta }}_{\rho }\right].$ Additionally, $F\left(t,\omega \left(t\right)\right)$ can be reformulated as $\left[{\underline{F}}_{\rho }\left(t,{\underline{\omega }}_{\rho }\left(t\right),{\bar{\omega }}_{\rho }\left(t\right)\right),\;\right.\left.{\bar{F}}_{\rho }\left(t,{\underline{\omega }}_{\rho }\left(t\right),{\bar{\omega }}_{\rho }\left(t\right)\right)\right]$. The following systems will hold based on using Theorem 3:

(1) If $\omega \left(t\right)$ is $\left(1;\beta \right)$-fuzzy conformable differentiable, then the corresponding crisp system of the IVPs (1) and (2) will be written in the form of the following:
  (3) $$\left\{\begin{array}{c} C^{\beta }{\underline{\omega }}_{\rho }\left(t\right)={\underline{F}}_{\rho }\left(t,{\underline{\omega }}_{\rho }\left(t\right),{\bar{\omega }}_{\rho }\left(t\right)\right)\\ C^{\beta }{\bar{\omega }}_{\rho }\left(t\right)={\bar{F}}_{\rho }\left(t,{\underline{\omega }}_{\rho }\left(t\right),{\bar{\omega }}_{\rho }\left(t\right)\right)\\ {\underline{\omega }}_{\rho }\left(a\right)={\underline{\delta }}_{\rho },\;\;{\bar{\omega }}_{\rho }\left(a\right)={\bar{\delta }}_{\rho } \end{array}\right.$$
(2) If $\omega \left(t\right)$ is $\left(2;\beta \right)$-fuzzy conformable differentiable, then the corresponding crisp system of IVPs (1) and (2) will be written in the form of the following:
  (4) $$\left\{\begin{array}{c} C^{\beta }{\underline{\omega }}_{\rho }\left(t\right)={\bar{F}}_{\rho }\left(t,{\underline{\omega }}_{\rho }\left(t\right),{\bar{\omega }}_{\rho }\left(t\right)\right)\\ C^{\beta }{\bar{\omega }}_{\rho }\left(t\right)={\underline{F}}_{\rho }\left(t,{\underline{\omega }}_{\rho }\left(t\right),{\bar{\omega }}_{\rho }\left(t\right)\right)\\ {\underline{\omega }}_{\rho }\left(a\right)={\underline{\delta }}_{\rho },\;\;{\bar{\omega }}_{\rho }\left(a\right)={\bar{\delta }}_{\rho } \end{array}\right.$$
  The formulation of the fuzzy fractional IVPs (1) and (2) along with Theorem 2.3 show us how to deal with numerical solutions of fuzzy fractional IVPs. The original fuzzy fractional IVPs can be converted into a crisp system of fractional IVPs equivalently. This indicates that no need to rewrite the numerical methods for the crisp systems of the fractional IVPs in the fuzzy setting, but, instead, we may use the numerical methods directly on the obtained crisp systems.

## 4. Primary Principle of Residual Power Series Approach

This section is devoted to justifying the strategy of our proposed method in predicting and investigating the approximate solutions for the fuzzy fractional IVPs (1) and (2). The basic mainstay of the RPS approach is applying the residual error notion and the fractional Taylors series, where the components of truncated fractional Taylor’s series are computed via deriving the truncated fractional residual functions [41,42,43,44,45,46,47,48,49], see also [50,51,52,53] for further results.

**Theorem** **5.** *For* $\rho \in \left[0,1\right]$ *, let* ${\underline{\omega }}_{\rho }\left(t\right)$ *, and* ${\bar{\omega }}_{\rho }\left(t\right)$ *have the following fractional expansions about* t=η *,*

(5) $$\begin{array}{c} {\underline{\omega }}_{\rho }\left(t\right)=\overset{\infty }{\underset{k=0}{\sum }}\frac{c_{k}t^{\beta k}}{\beta^{k}k!},\\ {\bar{\omega }}_{\rho }\left(t\right)=\overset{\infty }{\underset{k=0}{\sum }}\frac{d_{k}t^{\beta k}}{\beta^{k}k!}, \end{array}$$

*where* 0<β≤1 *and* $t\in \left[\;\eta ,\eta +R\right)$ *. If* $C^{\beta }{\underline{\omega }}_{\rho }\left(t\right)$ *and* $C^{\beta }{\bar{\omega }}_{\rho }\left(t\right)$ *are two continuous on* $\left[\;\eta ,\eta +R\right)$ *, then the unknown functions* $c_{k}$ *and* $d_{k}$ *are in the forms* $c_{k}=\frac{C^{k\beta }{\underline{\omega }}_{\rho }\left(\eta \right)}{\beta^{k}k!}$ *and* $d_{k}=\frac{C^{k\beta }{\bar{\omega }}_{\rho }\left(\eta \right)}{\beta^{k}k!}$ *for* k=0,1,2,…, *where* $C^{k\beta }=C^{\beta }\cdot C^{\beta }\cdots C^{\beta },$ *k-times.*

**Proof.**  We need to prove that the unknown coefficients in the fractional expansions (5) have the forms:

$$c_{k}=\frac{C^{k\beta }{\underline{\omega }}_{\rho }\left(\eta \right)}{\beta^{k}k!}\;\mathrm{and}\;d_{k}=\frac{C^{k\beta }{\bar{\omega }}_{\rho }\left(\eta \right)}{\beta^{k}k!}\;\mathrm{for}\;k=0,1,2,\ldots .$$

Suppose that ${\underline{\omega }}_{\rho }\left(t\right)$ and ${\bar{\omega }}_{\rho }\left(t\right)$ are two functions which have the fractional PS expansions as in Definition 2.5. Its clear that, if we put t=η in (5) leads to $c_{0}={\underline{\omega }}_{\rho }\left(\eta \right)$, $d_{0}={\bar{\omega }}_{\rho }\left(\eta \right)$ and $c_{k}=d_{k}=0$, for k≥1. Next, by operating β-th conformable fractional derivative on both sides of (5) gives

(6) $$\begin{array}{c} C^{\beta }{\underline{\omega }}_{\rho }\left(t\right)=\beta c_{1}+2\beta c_{2}{\left(t-\eta \right)}^{\beta }+3\beta c_{3}{\left(t-\eta \right)}^{2\beta }+4\beta c_{4}{\left(t-\eta \right)}^{3\beta }+\cdots ,\\ C^{\beta }{\bar{\omega }}_{\rho }\left(t\right)=\beta d_{1}+2\beta d_{2}{\left(t-\eta \right)}^{2\beta }+3\beta d_{3}{\left(t-\eta \right)}^{2\beta }+4\beta d_{4}{\left(t-\eta \right)}^{3\beta }+\cdots . \end{array}$$

Substitution of t=η into (6) leads to $c_{1}=\frac{C^{\beta }{\underline{\omega }}_{\rho }\left(\eta \right)}{\beta }$ and $d_{1}=\frac{C^{\beta }{\bar{\omega }}_{\rho }\left(\eta \right)}{\beta }$.

Additionally, we can apply $C^{\beta }$ on both sides of (6) to get

(7) $$\begin{array}{c} C^{2\beta }{\underline{\omega }}_{\rho }\left(t\right)=2\beta^{2}c_{2}+6\beta^{2}c_{3}{\left(t-\eta \right)}^{\beta }+12\beta^{2}c_{4}{\left(t-\eta \right)}^{2\beta }+\cdots ,\\ C^{2\beta }{\bar{\omega }}_{\rho }\left(t\right)=2\beta^{2}d_{2}+6\beta^{2}d_{3}{\left(t-\eta \right)}^{\beta }+12\beta^{2}d_{4}{\left(t-\eta \right)}^{2\beta }+\cdots . \end{array}$$

Then, by substituting t=η into (7) gives that $c_{2}=\frac{C^{2\beta }{\underline{\omega }}_{\rho }\left(\eta \right)}{2\beta^{2}}$ and $d_{2}=\frac{C^{2\beta }{\bar{\omega }}_{\rho }\left(\eta \right)}{2\beta^{2}}$.

Again, by operating $C^{\beta }$ on both sides of (7), we have

(8) $$\begin{array}{c} C^{3\beta }{\underline{\omega }}_{\rho }\left(t\right)=6\beta^{3}c_{3}+24\beta^{3}c_{4}{\left(t-\eta \right)}^{\beta }+\cdots ,\\ C^{3\beta }{\bar{\omega }}_{\rho }\left(t\right)=6\beta^{3}d_{3}+24\beta^{3}d_{4}{\left(t-\eta \right)}^{\beta }+\cdots . \end{array}$$

After that, substitute t=η into (8) to obtain that $c_{3}=\frac{C^{3\beta }{\underline{\omega }}_{\rho }\left(\eta \right)}{3!\beta^{3}}$ and $d_{3}=\frac{C^{3\beta }{\bar{\omega }}_{\rho }\left(\eta \right)}{3!\beta^{3}}$. Continuing in the same manner, apply $C^{\beta }$ k-times, and then substitute t=η into the obtained fractional expansions so that the pattern of $c_{k}$ and $d_{k}$ can be found. Therefore, the unknown coefficients in the fractional expansions (5) have the forms

$$c_{k}=\frac{C^{k\beta }{\underline{\omega }}_{\rho }\left(\eta \right)}{\beta^{k}k!}\;\mathrm{and}\;d_{k}=\frac{C^{k\beta }{\bar{\omega }}_{\rho }\left(\eta \right)}{\beta^{k}k!}\;\mathrm{for}\;k=0,1,2,\ldots$$

Now, the process of obtaining $\left(1\right)$-solution of the crisp system (3) corresponding the first case of fuzzy fractional IVPs (1) and (2) will be discussed. The same fashion can be used to create $\left(2\right)$-solution. To reach our purpose, we assume that the solutions of the crisp system (3) about the initial point t=0 have the following fractional PS forms

(9) $$\begin{array}{c} {\underline{\omega }}_{\rho }\left(t\right)=\overset{\infty }{\underset{k=0}{\sum }}\frac{c_{k}t^{\beta k}}{\beta^{k}k!},\;t\geq 0,\;0<\beta \leq 1,\;\rho \in \left[0,1\right],\\ {\bar{\omega }}_{\rho }\left(t\right)=\overset{\infty }{\underset{k=0}{\sum }}\frac{d_{k}t^{\beta k}}{\beta^{k}k!},\;t\geq 0,\;0<\beta \leq 1,\;\rho \in \left[0,1\right]. \end{array}$$

We can approximate the solutions ${\underline{\omega }}_{\rho }\left(t\right)$ and ${\bar{\omega }}_{\rho }\left(t\right)$ for the system (3) by the following j-th fractional PS approximate solutions

(10) $$\begin{array}{c} {\underline{\omega }}_{\rho }^{j}\left(t\right)=\overset{j}{\underset{k=0}{\sum }}\frac{c_{k}t^{\beta k}}{\beta^{k}k!},\;t\geq 0,\;0<\beta \leq 1,\;\rho \in \left[0,1\right],\\ {\bar{\omega }}_{\rho }^{j}\left(t\right)=\overset{j}{\underset{k=0}{\sum }}\frac{d_{k}t^{\beta k}}{\beta^{k}k!},\;t\geq 0,\;0<\beta \leq 1,\;\rho \in \left[0,1\right]. \end{array}$$

Applying the initial data of (3), when j=0, in the expansions (10), we verify that the 0-th fractional PS approximate solutions of ${\underline{\omega }}_{\rho }\left(t\right)$ and ${\bar{\omega }}_{\rho }\left(t\right)$ are ${\underline{\omega }}_{\rho }^{0}\left(t\right)=c_{0}={\underline{\delta }}_{\rho }={\underline{\omega }}_{\rho }\left(0\right)$ and ${\bar{\omega }}_{\rho }^{j}\left(t\right)=d_{0}={\bar{\delta }}_{\rho }={\bar{\omega }}_{\rho }\left(0\right)$.

Hence, the j-th fractional PS approximate solutions (10) can be reformulated as

(11) $$\begin{array}{c} {\underline{\omega }}_{\rho }^{j}\left(t\right)={\underline{\delta }}_{\rho }+\overset{j}{\underset{k=1}{\sum }}\frac{c_{k}t^{\beta k}}{\beta^{k}k!},\;t\geq 0,\;0<\beta \leq 1,\;\rho \in \left[0,1\right],\\ {\bar{\omega }}_{\rho }^{j}\left(t\right)={\bar{\delta }}_{\rho }+\overset{j}{\underset{k=0}{\sum }}\frac{d_{k}t^{\beta k}}{\beta^{k}k!},\;t\geq 0,\;0<\beta \leq 1,\;\rho \in \left[0,1\right]. \end{array}$$

To find out the coefficients $c_{k}$ and $d_{k}$, for k=1,2,3,…,j of the fractional expansions (11), one can solve the following fractional algebraic equations manually for the target coefficients

(12) $$\begin{array}{c} C^{\left(j-1\right)\beta }{\underline{Res}}_{\rho }^{j}\left(0\right)=0,\\ C^{\left(j-1\right)\beta }{\bar{Res}}_{\rho }^{j}\left(0\right)=0,\;j=1,2,\ldots , \end{array}$$

where ${\underline{Res}}_{\rho }^{j}$ and ${\bar{Res}}_{\rho }^{j}$ are called the j-th fractional residual functions of the crisp system (3) and defined as follows

(13) $$\begin{array}{c} {\underline{Res}}_{\rho }^{j}\left(t\right)=C^{\beta }{\underline{\omega }}_{\rho }^{j}\left(t\right)-{\underline{F}}_{\rho }\left(t,{\underline{\omega }}_{\rho }^{j}\left(t\right),{\bar{\omega }}_{\rho }^{j}\left(t\right)\right),\\ {\bar{Res}}_{\rho }^{j}\left(t\right)=C^{\beta }{\bar{\omega }}_{\rho }^{j}\left(t\right)-{\bar{F}}_{\rho }\left(t,{\underline{\omega }}_{\rho }^{j}\left(t\right),{\bar{\omega }}_{\rho }^{j}\left(t\right)\right), \end{array}$$

and the ∞-th fractional residual functions of the system (3) have the forms

(14) $$\begin{array}{c} \underset{j\to \infty }{\lim}{\underline{Res}}_{\rho }^{j}\left(t\right)={\underline{Res}}_{\rho }\left(t\right)=C^{\beta }{\underline{\omega }}_{\rho }\left(t\right)-{\underline{F}}_{\rho }\left(t,{\underline{\omega }}_{\rho }\left(t\right),{\bar{\omega }}_{\rho }\left(t\right)\right),\\ \underset{j\to \infty }{\lim}{\bar{Res}}_{\rho }^{j}\left(t\right)={\bar{Res}}_{\rho }\left(t\right)=C^{\beta }{\bar{\omega }}_{\rho }\left(t\right)-{\bar{F}}_{\rho }\left(t,{\underline{\omega }}_{\rho }\left(t\right),{\bar{\omega }}_{\rho }\left(t\right)\right). \end{array}$$

Indeed, some useful facts concerned with the fractional residual functions are listed below, and form the mainstay of the RPS scheme

▪ ${\underline{Res}}_{\rho }\left(t\right)=0$ and ${\bar{Res}}_{\rho }\left(t\right)=0$ for each t≥0.
▪ $\underset{j\to \infty }{\lim}{\underline{Res}}_{\rho }^{j}\left(t\right)={\underline{Res}}_{\rho }\left(t\right)$ and $\underset{j\to \infty }{\lim}{\bar{Res}}_{\rho }^{j}\left(t\right)={\bar{Res}}_{\rho }\left(t\right)$ for each t≥0.
▪ $C^{m\beta }{\underline{Res}}_{\rho }^{j}\left(0\right)=0$ and $C^{m\beta }{\bar{Res}}_{\rho }^{j}\left(0\right)$ for m=0,1,2,…,j.

Based on this analysis, the process of obtaining the coefficients $c_{k}$ and $d_{k}$ in the fractional expansions (11) construct the fractional PS approximate solutions for the system (3) by RPS method which can be summarized via the next algorithm. □

**Algorithm 1.** To deduce the approximate solutions of (3) in detail, one can perform the following manner by one of the known software packages like MATHEMATICA 12.**Step I:** Write the system (3) in the form
$$\left\{\begin{array}{c} C^{\beta }{\underline{\omega }}_{\rho }\left(t\right)-{\underline{F}}_{\rho }\left(t,{\underline{\omega }}_{\rho }\left(t\right),{\bar{\omega }}_{\rho }\left(t\right)\right)=0\\ C^{\beta }{\bar{\omega }}_{\rho }\left(t\right)-{\bar{F}}_{\rho }\left(t,{\underline{\omega }}_{\rho }\left(t\right),{\bar{\omega }}_{\rho }\left(t\right)\right)=0 \end{array}\right.$$**Step II:** Suppose that the solutions of the system (3) about the initial point t=0 have the fractional PS expansion forms
$${\underline{\omega }}_{\rho }\left(t\right)=\overset{\infty }{\underset{k=0}{\sum }}\frac{c_{k}t^{\beta k}}{\beta^{k}k!},{\bar{\omega }}_{\rho }\left(t\right)=\overset{\infty }{\underset{k=0}{\sum }}\frac{d_{k}t^{\beta k}}{\beta^{k}k!},\;t\geq 0,\;0<\beta \leq 1,\;\rho \in \left[0,1\right].$$**Step III:** Set $c_{0}={\underline{\omega }}_{\rho }\left(0\right)={\underline{\delta }}_{\rho }$ and $d_{0}={\bar{\omega }}_{\rho }\left(0\right)={\bar{\delta }}_{\rho }$, then the j-th fractional PS approximate solutions ${\underline{\omega }}_{\rho }^{j}\left(t\right)$ and ${\bar{\omega }}_{\rho }^{j}\left(t\right)$ of ${\underline{\omega }}_{\rho }\left(t\right)\;\mathrm{and}\;{\bar{\omega }}_{\rho }\left(t\right)$ can be written respectively as
$${\underline{\omega }}_{\rho }^{j}\left(t\right)=c_{0}+\overset{j}{\underset{k=1}{\sum }}\frac{c_{k}t^{\beta k}}{\beta^{k}k!}\;\mathrm{and}\;{\bar{\omega }}_{\rho }^{j}\left(t\right)=d_{0}+\overset{j}{\underset{k=1}{\sum }}\frac{d_{k}t^{\beta k}}{\beta^{k}k!},t\geq 0,\;0<\beta \leq 1,\;\rho \in \left[0,1\right].$$**Step IV:** Define the j-th fractional residual functions ${\underline{Res}}_{\rho }^{j}\left(t\right)$ and ${\bar{Res}}_{\rho }^{j}\left(t\right)$ such that
$$\begin{array}{c} {\underline{Res}}_{\rho }^{j}\left(t\right)=C^{\beta }{\underline{\omega }}_{\rho }^{j}\left(t\right)-{\underline{F}}_{\rho }\left(t,{\underline{\omega }}_{\rho }^{j}\left(t\right),{\bar{\omega }}_{\rho }^{j}\left(t\right)\right),\\ {\bar{Res}}_{\rho }^{j}\left(t\right)=C^{\beta }{\bar{\omega }}_{\rho }^{j}\left(t\right)-{\bar{F}}_{\rho }\left(t,{\underline{\omega }}_{\rho }^{j}\left(t\right),{\bar{\omega }}_{\rho }^{j}\left(t\right)\right). \end{array}$$**Step V:** Substitute ${\underline{\omega }}_{\rho }^{j}\left(t\right)$ and ${\bar{\omega }}_{\rho }^{j}\left(t\right)$ in ${\underline{Res}}_{\rho }^{j}\left(t\right)$ and ${\bar{Res}}_{\rho }^{j}\left(t\right)$ so that
$$\begin{array}{c} {\underline{Res}}_{\rho }^{j}\left(t\right)=C^{\beta }\left(c_{0}+\overset{j}{\underset{k=1}{\sum }}\frac{c_{k}t^{\beta k}}{\beta^{k}k!}\right)-{\underline{F}}_{\rho }\left(t,c_{0}+\overset{j}{\underset{k=1}{\sum }}\frac{c_{k}t^{\beta k}}{\beta^{k}k!},d_{0}+\overset{j}{\underset{k=0}{\sum }}\frac{d_{k}t^{\beta k}}{\beta^{k}k!}\right),\\ {\bar{Res}}_{\rho }^{j}\left(t\right)=C^{\beta }\left(d_{0}+\overset{j}{\underset{k=0}{\sum }}\frac{d_{k}t^{\beta k}}{\beta^{k}k!}\right)-{\bar{F}}_{\rho }\left(t,c_{0}+\overset{j}{\underset{k=1}{\sum }}\frac{c_{k}t^{\beta k}}{\beta^{k}k!},d_{0}+\overset{j}{\underset{k=0}{\sum }}\frac{d_{k}t^{\beta k}}{\beta^{k}k!}\right). \end{array}$$**Step VI:** Consider j=1, in **Step V**, then solve ${\underline{Res}}_{\rho }^{1}\left(t\right)=0$ and ${\bar{Res}}_{\rho }^{1}\left(t\right)=0$ at t=0 for $c_{1}$ and $d_{1}$. Therefore, the first fractional PS approximate solutions ${\underline{\omega }}_{\rho }^{1}\left(t\right)$ and ${\bar{\omega }}_{\rho }^{1}\left(t\right)$ will be obtained.**Step VII:** For j=2,3,…,r in **Step V**, apply the operator $\left(j-1\right)\beta$-th on both sides of the resulting fractional equations such that $C^{\left(j-1\right)\beta }{\underline{Res}}_{\rho }^{j}\left(t\right)$ and $C^{\left(j-1\right)\beta }{\bar{Res}}_{\rho }^{j}\left(t\right)$. Then, by solving $C^{\left(j-1\right)\beta }{\underline{Res}}_{\rho }^{j}\left(0\right)=0$ and $C^{\left(j-1\right)\beta }{\bar{Res}}_{\rho }^{j}\left(0\right)=0$, $c_{j}$ and $d_{j}$ can be obtained.**Step VIII:** Write the forms of the obtained coefficients $c_{j}$ and $d_{j}$ in terms of j-th fractional PS expansions ${\underline{\omega }}_{\rho }^{j}\left(t\right)$ and ${\bar{\omega }}_{\rho }^{j}\left(t\right)$ and repeat the above steps to reach a closed-form in terms of infinite series as in **Step II**. Elsewhere, the solution obtained will be representing the j-th fractional PS approximate solutions of the crisp system (3).

## 5. Applications and Numerical Simulations

In this section, we consider two fuzzy fractional IVPs of order β to demonstrate the efficiency and applicability of the RPS approach. Here, all of the symbolic and numerical computations performed by using Mathematica 12.

**Application 1.** Consider the following fuzzy fractional IVPs

(15) $$C^{\beta }\omega \left(t\right)=\left[\rho +1,3-\rho \right]+\omega \left(t\right)\;,t\in \left[0,1\right],$$

with the fuzzy initial condition

(16) $$\omega \left(0\right)=0,$$

where 0<β≤1 and $\rho \in \left[0,1\right]$.

By using Theorem 3 and the type of fuzzy conformable differentiability, we have the following cases:

**Case I:** If $\omega \left(t\right)$ is $\left(1;\beta \right)$-fuzzy conformable differentiable, then the corresponding crisp system of the fuzzy fractional IVPs (15) and (16) will be written in the form of the following:

(17) $$\left\{\begin{array}{c} C^{\beta }{\underline{\omega }}_{\rho }\left(t\right)=\left(\rho +1\right)+{\underline{\omega }}_{\rho }\left(t\right),\\ C^{\beta }{\bar{\omega }}_{\rho }\left(t\right)=\left(3-\rho \right)+{\bar{\omega }}_{\rho }\left(t\right),\\ {\underline{\omega }}_{\rho }\left(0\right)=0,\;\;\;\;{\bar{\omega }}_{\rho }\left(0\right)=0, \end{array}\right.$$

For the standard case β=1, the fuzzy exact solution in the ρ-cut representation has the form ${\left[\omega \left(t\right)\right]}_{\rho }=\left[\rho +1,3-\rho \right]\left(e^{t}-1\right).$

In view of the last discussion for the RPS scheme, starting with ${\underline{\omega }}_{\rho }\left(0\right)=0$ and ${\bar{\omega }}_{\rho }\left(0\right)=0$, assume that the j-th approximate fractional PS solutions for the fractional IVPs system (17) have the following forms

(18) $${\underline{\omega }}_{\rho }^{j}\left(t\right)=\overset{j}{\underset{k=1}{\sum }}\frac{c_{k}t^{\beta k}}{\beta^{k}k!},{\bar{\omega }}_{\rho }^{j}\left(t\right)=\overset{j}{\underset{k=0}{\sum }}\frac{d_{k}t^{\beta k}}{\beta^{k}k!},t\geq 0,\;0<\beta \leq 1,\;\rho \in \left[0,1\right],$$

where the unknown coefficients $c_{k}$ and $d_{k}$ for k=1,2, 3,…,j can be determined by constructing the j-th fractional residual functions ${\underline{Res}}_{\rho }^{j}\left(t\right)$ and ${\bar{Res}}_{\rho }^{j}\left(t\right)$ for (17) such that

(19) $$\begin{array}{c} {\underline{Res}}_{\rho }^{j}\left(t\right)=C^{\beta }\left(\overset{j}{\underset{k=1}{\sum }}\frac{c_{k}t^{\beta k}}{\beta^{k}k!},\right)-\overset{j}{\underset{k=1}{\sum }}\frac{c_{k}t^{\beta k}}{\beta^{k}k!}-\left(\rho +1\right),\\ {\bar{Res}}_{\rho }^{j}\left(t\right)=C^{\beta }\left(\overset{j}{\underset{k=1}{\sum }}\frac{d_{k}t^{\beta k}}{\beta^{k}k!}\right)-\overset{j}{\underset{k=0}{\sum }}\frac{d_{k}t^{\beta k}}{\beta^{k}k!}-\left(3-\rho \right). \end{array}$$

For j=1, we have ${\underline{Res}}_{\rho }^{1}\left(t\right)=C^{\beta }\left(\frac{c_{1}t^{\beta }}{\beta }\right)-\frac{c_{1}t^{\beta }}{\beta }-\left(\rho +1\right)=c_{1}\left(\frac{\beta -t^{\beta }}{\beta }\right)-\rho -1$ and ${\bar{Res}}_{\rho }^{1}\left(t\right)=C^{\beta }\left(\frac{d_{1}t^{\beta }}{\beta }\right)-\frac{d_{1}t^{\beta }}{\beta }-\left(3-\rho \right)=d_{1}\left(\frac{\beta -t^{\beta }}{\beta }\right)-3+\rho$. Then, ${\underline{Res}}_{\rho }^{1}\left(0\right)=0$ and ${\bar{Res}}_{\rho }^{1}\left(0\right)=0$ gives $c_{1}=\rho +1$ and $d_{1}=3-\rho$.

For j=2, we have ${\underline{Res}}_{\rho }^{2}\left(t\right)=C^{\beta }\left(\frac{\left(\rho +1\right)t^{\beta }}{\beta }+\frac{c_{2}t^{2\beta }}{2\beta^{2}}\right)-\left(\frac{\left(\rho +1\right)t^{\beta }}{\beta }+\frac{c_{2}t^{2\beta }}{2\beta^{2}}\right)-\left(\rho +1\right)=\left(\left(\rho +1\right)+\frac{c_{2}t^{\beta }}{\beta }\right)-\left(\frac{\left(\rho +1\right)t^{\beta }}{\beta }+\frac{c_{2}t^{2\beta }}{2\beta^{2}}\right)-\rho -1$ and ${\bar{Res}}_{\rho }^{2}\left(t\right)=C^{\beta }\left(\frac{\left(3-\rho \right)t^{\beta }}{\beta }+\frac{d_{2}t^{2\beta }}{2\beta^{2}}\right)-\left(\frac{d_{1}t^{\beta }}{\beta }+\frac{d_{2}t^{2\beta }}{2\beta^{2}}\right)-\left(3-\rho \right)=\left(\left(3-\rho \right)+\frac{d_{2}t^{\beta }}{\beta }\right)-\left(\frac{\left(3-\rho \right)t^{\beta }}{\beta }+\frac{d_{2}t^{2\beta }}{2\beta^{2}}\right)-3+\rho .$ By applying $C^{\beta }$ both sides of ${\underline{Res}}_{\rho }^{2}\left(t\right)$ and ${\bar{Res}}_{\rho }^{2}\left(t\right)$ yields $C^{\beta }{\underline{Res}}_{\rho }^{2}\left(t\right)=c_{2}-\left(\rho +1\right)-\frac{c_{2}t^{\beta }}{\beta }$ and $C^{\beta }{\bar{Res}}_{\rho }^{2}\left(t\right)=d_{2}-\left(3-\rho \right)-\frac{d_{2}t^{\beta }}{\beta }$ and then, by solving ${\underline{Res}}_{\rho }^{2}\left(0\right)=0$ and ${\bar{Res}}_{\rho }^{2}\left(0\right)=0$, we conclude that $c_{2}=\rho +1$, and $d_{2}=3-\rho$.

In the same manner, for j=3, we have $C^{2\beta }{\underline{Res}}_{\rho }^{3}\left(t\right)=C^{2\beta }\left(C^{\beta }\left(\frac{\left(\rho +1\right)t^{\beta }}{\beta }+\frac{\left(\rho +1\right)t^{2\beta }}{2\beta^{2}}+\frac{c_{3}t^{3\beta }}{6\beta^{3}}\right)-\left(\frac{\left(\rho +1\right)t^{\beta }}{\beta }+\frac{\left(\rho +1\right)t^{2\beta }}{2\beta^{2}}+\frac{c_{3}t^{3\beta }}{6\beta^{3}}\right)-\left(\rho +1\right)\right)=C^{2\beta }\left(\left(\left(\rho +1\right)+\frac{\left(\rho +1\right)t^{\beta }}{\beta }+\frac{c_{3}t^{2\beta }}{2\beta^{2}}\right)-\left(\frac{\left(\rho +1\right)t^{\beta }}{\beta }+\frac{\left(\rho +1\right)t^{2\beta }}{2\beta^{2}}+\frac{c_{3}t^{3\beta }}{6\beta^{3}}\right)-\rho -1\right)=c_{3}-\left(\rho +1\right)-\frac{c_{3}t^{\beta }}{2\beta }$ and $C^{2\beta }{\bar{Res}}_{\rho }^{3}\left(t\right)=C^{2\beta }\left(C^{\beta }\left(\frac{\left(3-\rho \right)t^{\beta }}{\beta }+\frac{\left(3-\rho \right)t^{2\beta }}{2\beta^{2}}+\frac{d_{3}t^{3\beta }}{6\beta^{3}}\right)-\left(\frac{\left(3-\rho \right)t^{\beta }}{\beta }+\frac{\left(3-\rho \right)t^{2\beta }}{2\beta^{2}}+\frac{d_{3}t^{3\beta }}{6\beta^{3}}\right)-\left(3-\rho \right)\right)=C^{2\beta }\left(\left(\left(3-\rho \right)+\frac{\left(3-\rho \right)t^{\beta }}{\beta }+\frac{d_{3}t^{2\beta }}{2\beta^{2}}\right)-\left(\frac{\left(3-\rho \right)t^{\beta }}{\beta }+\frac{\left(3-\rho \right)t^{2\beta }}{2\beta^{2}}+\frac{d_{3}t^{3\beta }}{6\beta^{3}}\right)-\rho +3\right)=d_{3}-\left(3-\rho \right)-\frac{d_{3}t^{\beta }}{2\beta }$. Thus, by using the fact that $C^{2\beta }{\underline{Res}}_{\rho }^{3}\left(0\right)=0$ and $C^{2\beta }{\bar{Res}}_{\rho }^{3}\left(0\right)=0$, it yields that $c_{3}=\rho +1$ and $d_{3}=3-\rho$.

Continuing in this procedure, based upon the fact $C^{\left(j-1\right)\beta }{\underline{Res}}_{\rho }^{j}\left(0\right)=0$ and $C^{\left(j-1\right)\beta }{\bar{Res}}_{\rho }^{j}\left(0\right)=0,$ for j=4,5,6,…, it can be concluded that $c_{j}=\rho +1$ and $d_{j}=3-\rho$. Therefore, the j-th fractional PS expansions of the fractional IVPs (17) could be expressed as:

(20) $$\begin{array}{c} {\underline{\omega }}_{\rho }^{j}\left(t\right)=\frac{\left(\rho +1\right)t^{\beta }}{\beta }+\frac{\left(\rho +1\right)t^{2\beta }}{\beta^{2}2!}+\frac{\left(\rho +1\right)t^{3\beta }}{\beta^{3}3!}+\cdots +\frac{\left(\rho +1\right)t^{j\beta }}{\beta^{j}j!},\\ {\bar{\omega }}_{\rho }^{j}\left(t\right)=\frac{\left(3-\rho \right)t^{\beta }}{\beta }+\frac{\left(3-\rho \right)t^{2\beta }}{\beta^{2}2!}+\frac{\left(3-\rho \right)t^{3\beta }}{\beta^{3}3!}+\cdots +\frac{\left(3-\rho \right)t^{j\beta }}{\beta^{j}j!}. \end{array}$$

Moreover, the fractional PS approximate solutions of the fractional IVPs (17) have the general form in terms of the infinite series

(21) $$\begin{array}{c} {\underline{\omega }}_{\rho }\left(t\right)=\frac{\left(\rho +1\right)t^{\beta }}{\beta }+\frac{\left(\rho +1\right)t^{2\beta }}{\beta^{2}2!}+\frac{\left(\rho +1\right)t^{3\beta }}{\beta^{3}3!}+\cdots =\left(\rho +1\right)\overset{\infty }{\underset{k=0}{\sum }}\frac{t^{k\beta }}{\beta^{k}k!},\;\\ {\bar{\omega }}_{\rho }\left(t\right)=\frac{\left(3-\rho \right)t^{\beta }}{\beta }+\frac{\left(3-\rho \right)t^{2\beta }}{\beta^{2}2!}+\frac{\left(3-\rho \right)t^{3\beta }}{\beta^{3}3!}+\cdots =\left(3-\rho \right)\overset{\infty }{\underset{k=0}{\sum }}\frac{t^{k\beta }}{\beta^{k}k!}. \end{array}$$

In particular, for β=1 in (21), we have

(22) $$\begin{array}{c} {\underline{\omega }}_{\rho }\left(t\right)=\left(\rho +1\right)\left(t+\frac{t^{2}}{2!}+\frac{t^{3}}{3!}+\cdots \right)=\left(\rho +1\right)\overset{\infty }{\underset{k=0}{\sum }}\frac{t^{k}}{k!}\;,\\ {\bar{\omega }}_{\rho }\left(t\right)=\left(3-\rho \right)\left(t+\frac{t^{2}}{2!}+\frac{t^{3}}{3!}+\cdots \right)=\left(3-\rho \right)\overset{\infty }{\underset{k=0}{\sum }}\frac{t^{k}}{k!}, \end{array}$$

which are compatible with the McLaurin series of the fuzzy exact solution ${\left[\omega \left(t\right)\right]}_{\rho }=\left[\rho +1,3-\rho \right]\left(e^{t}-1\right).$

**Case II:** If $\omega \left(t\right)$ is $\left(2;\beta \right)$-fuzzy conformable differentiable, then the corresponding crisp system of fuzzy fractional IVPs (15) and (16) will be written in the form of the following

(23) $$\left\{\begin{array}{c} C^{\beta }{\underline{\omega }}_{\rho }\left(t\right)=\left(3-\rho \right)+{\bar{\omega }}_{\rho }\left(t\right),\\ C^{\beta }{\bar{\omega }}_{\rho }\left(t\right)=\left(\rho +1\right)+{\underline{\omega }}_{\rho }\left(t\right),\\ {\underline{\omega }}_{\rho }\left(0\right)=0,\;\;\;\;{\bar{\omega }}_{\rho }\left(0\right)=0. \end{array}\right.$$

For the standard case β=1, the fuzzy exact solution in term of ρ-cut representation has the form ${\left[\omega \left(t\right)\right]}_{\rho }=2e^{t}+\left[1-\rho ,\rho -1\right]\left(1-e^{-t}\right).$

According to RPS procedure, the j-th fractional residual functions ${\underline{Res}}_{\rho }^{j}\left(t\right)$ and ${\bar{Res}}_{\rho }^{j}\left(t\right)$ of the fractional IVPs (23) could be written as:

(24) $$\begin{array}{c} {\underline{Res}}_{\rho }^{j}\left(t\right)=C^{\beta }\left({\underline{\omega }}_{\rho }^{j}\left(t\right)\right)-{\bar{\omega }}_{\rho }^{j}\left(t\right)-\left(3-\rho \right),\\ {\bar{Res}}_{\rho }^{j}\left(t\right)=C^{\beta }\left({\bar{\omega }}_{\rho }^{j}\left(t\right)\right)-{\underline{\omega }}_{\rho }^{j}\left(t\right)-\left(\rho +1\right), \end{array}$$

where ${\underline{\omega }}_{\rho }^{j}\left(t\right)$ and ${\bar{\omega }}_{\rho }^{j}\left(t\right)$ represent to the j-th fractional PS approximate solutions of (23) such that

(25) $${\underline{\omega }}_{\rho }^{j}\left(t\right)=\overset{j}{\underset{k=1}{\sum }}\frac{c_{k}t^{\beta k}}{\beta^{k}k!},{\bar{\omega }}_{\rho }^{j}\left(t\right)=\overset{j}{\underset{k=1}{\sum }}\frac{d_{k}t^{\beta k}}{\beta^{k}k!}.\;$$

Following the process of Algorithm 1, the values of $c_{k}$ and $d_{k}$, k=1,2,3,…,j, in fractional expansions (25) can be reached as follows,

$$c_{1}=3-\rho ,\;\;\;\;\;d_{1}=\rho +1,$$

$$c_{2}=\rho +1,\;\;\;\;\;d_{2}=3-\rho ,$$

$$c_{3}=3-\rho ,\;\;\;\;\;d_{3}=\rho +1,$$

$$c_{4}=\rho +1,\;\;\;d_{4}=3-\rho ,$$

$$c_{5}=3-\rho ,\;\;\;d_{5}=\rho +1,$$

$$c_{6}=\rho +1,\;\;\;d_{6}=3-\rho ,$$

⋮              ⋮

$$c_{j-1}=3-\rho ,\;\;d_{j-1}=\rho +1,$$

$$c_{j}=\rho +1,\;\;\;d_{j}=3-\rho .$$

Thus, the j-th fractional PS approximate solutions of fractional IVPs (23) have the expansions form

(26) $$\begin{array}{c} {\underline{\omega }}_{\rho }^{j}\left(t\right)=\frac{\left(3-\rho \right)t^{\beta }}{\beta }+\frac{\left(\rho +1\right)t^{2\beta }}{\beta^{2}2!}+\frac{\left(3-\rho \right)t^{3\beta }}{\beta^{3}3!}+\cdots +\frac{\left(3-\rho \right)t^{\left(j-1\right)\beta }}{\beta^{\left(j-1\right)}\left(j-1\right)!}+\frac{\left(\rho +1\right)t^{j\beta }}{\beta^{j}j!},\\ {\bar{\omega }}_{\rho }^{j}\left(t\right)=\frac{\left(\rho +1\right)t^{\beta }}{\beta }+\frac{\left(3-\rho \right)t^{2\beta }}{\beta^{2}2!}+\frac{\left(\rho +1\right)t^{3\beta }}{\beta^{3}3!}+\cdots +\frac{\left(\rho +1\right)t^{\left(j-1\right)\beta }}{\beta^{\left(j-1\right)}\left(j-1\right)!}+\frac{\left(3-\rho \right)t^{j\beta }}{\beta^{j}j!}. \end{array}$$

Correspondingly, the general forms of fractional PS approximate solutions of fractional IVPs (23) could be reformulated as

(27) $$\begin{array}{c} {\underline{\omega }}_{\rho }\left(t\right)=\left(3-\rho \right)\overset{\infty }{\underset{k=1}{\sum }}\frac{t^{\left(2k-1\right)\beta }}{\beta^{\left(2k-1\right)}\left(2k-1\right)!}+\left(\rho +1\right)\overset{\infty }{\underset{k=1}{\sum }}\frac{t^{2k\beta }}{\beta^{2k}\left(2k\right)!},\\ {\bar{\omega }}_{\rho }\left(t\right)=\left(\rho +1\right)\overset{\infty }{\underset{k=1}{\sum }}\frac{t^{\left(2k-1\right)\beta }}{\beta^{\left(2k-1\right)}\left(2k-1\right)!}+\left(3-\rho \right)\overset{\infty }{\underset{k=1}{\sum }}\frac{t^{2k\beta }}{\beta^{2k}\left(2k\right)!}, \end{array}$$

which agrees with the McLaurin series of the fuzzy exact solutions ${\left[\omega \left(t\right)\right]}_{\rho }=2e^{t}+\left[1-\rho ,\rho -1\right]\left(1-e^{-t}\right).$

**Figure 1:**
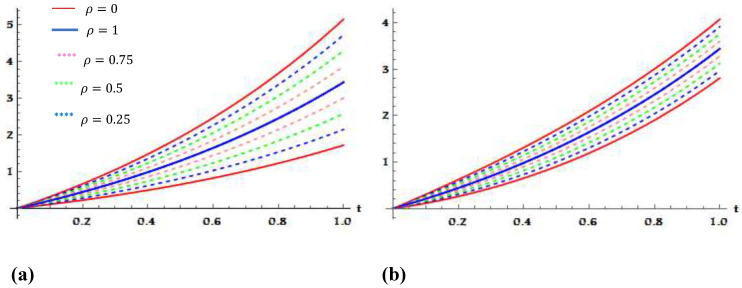
(**a**) Plots of ρ-cut representations of fuzzy exact solution $\left[{\underline{\omega }}_{\rho }\left(t\right),{\bar{\omega }}_{\rho }\left(t\right)\right]$ and fuzzy approximate solution $\left[{\underline{\omega }}_{\rho }^{8}\left(t\right),{\bar{\omega }}_{\rho }^{8}\left(t\right)\right]$, case I. (**b**) Plots of ρ-cut representations of fuzzy exact solution $\left[{\underline{\omega }}_{\rho }\left(t\right),{\bar{\omega }}_{\rho }\left(t\right)\right]$ and fuzzy approximate solution $\left[{\underline{\omega }}_{\rho }^{8}\left(t\right),{\bar{\omega }}_{\rho }^{8}\left(t\right)\right]$, case II, for Application 1 at β=1, $t\in \left[0,1\right]$.

The accuracy and efficiency of the RPS method are validated by calculating the absolute errors $E_{8}\left({\underline{\omega }}_{\rho }\right)=\left|{\underline{\omega }}_{\rho }\left(t\right)-{\underline{\omega }}_{\rho }^{8}\left(t\right)\right|$ and $E_{8}\left({\bar{\omega }}_{\rho }\right)=\left|{\bar{\omega }}_{\rho }\left(t\right)-{\bar{\omega }}_{\rho }^{8}\left(t\right)\right|$ for β=1 and different values of ρ, with some selected grid points of 0≤t≤1 as shown in Table 1 and Table 2. Graphically, to illustrate the effects of the parameter ρ on the behaviour of the fuzzy solutions, the exact and eighth fractional PS approximate solutions are plotted in Figure 1 at various values of ρ, where $\rho \in \left\{0,0.25,0.5,0.75,1\right\}$.

**Application 2.** Consider the following fuzzy fractional IVPs

(28) $$C^{\beta }\omega \left(t\right)=2t^{\beta }\omega \left(t\right)+\left[\rho -1,1-\rho \right]t^{\beta },\;t\in \left[0,1\right]$$

with the fuzzy initial condition

(29) $$\omega \left(0\right)=\left[\rho -1,1-\rho \right],$$

where 0<β≤1 and $\rho \in \left[0,1\right]$.

Using Theorem 3 based on the type of conformable differentiability, we have the following cases.

**Case I:** If $\omega \left(t\right)$ is $\left(1;\beta \right)$-fuzzy conformable differentiable, then the corresponding crisp system of the fuzzy fractional IVPs (28) and (29) can be written in the following form:

(30) $$\left\{\begin{array}{c} C^{\beta }{\underline{\omega }}_{\rho }\left(t\right)=2t^{\beta }{\underline{\omega }}_{\rho }\left(t\right)+\left(\rho -1\right)t^{\beta },\\ C^{\beta }{\bar{\omega }}_{\rho }\left(t\right)=2t^{\beta }{\bar{\omega }}_{\rho }\left(t\right)+\left(1-\rho \right)t^{\beta },\\ {\underline{\omega }}_{\rho }\left(0\right)=\rho -1,\;\;\;\;{\bar{\omega }}_{\rho }\left(0\right)=1-\rho . \end{array}\right.$$

For the standard case β=1, the fuzzy exact solution in the ρ-cut representation has the form ${\left[\omega \left(t\right)\right]}_{\rho }=\frac{1}{2}\left[\rho -1,1-\rho \right]\left(3e^{t^{2}}-1\right).$

As we mentioned earlier, set the zeroth approximate solutions of ${\underline{\omega }}_{\rho }\left(t\right)$, ${\bar{\omega }}_{\rho }\left(t\right)$, respectively, where ${\underline{\omega }}_{\rho }^{0}\left(t\right)=\rho -1$ and ${\bar{\omega }}_{\rho }^{0}\left(t\right)=1-\rho$, then the j-th fractional PS approximate solutions of the fractional IVPs (30) have the forms

(31) $$\begin{array}{c} {\underline{\omega }}_{\rho }^{j}\left(t\right)=\left(\rho -1\right)+\overset{j}{\underset{k=1}{\sum }}\frac{c_{k}t^{\beta k}}{\beta^{k}k!},\\ {\bar{\omega }}_{\rho }^{j}\left(t\right)=\left(1-\rho \right)+\overset{j}{\underset{k=0}{\sum }}\frac{d_{k}t^{\beta k}}{\beta^{k}k!}. \end{array}$$

To determine the values of the components $c_{k}$ and $d_{k}$, for k=1,2,3,…,j, solve the systems $C^{\left(j-1\right)\beta }{\underline{Res}}_{\rho }^{j}\left(t\right)=0$ and $C^{\left(j-1\right)\beta }{\bar{Res}}_{\rho }^{j}\left(t\right)=0$ at t=0 in which ${\underline{Res}}_{\rho }^{j}\left(t\right)$ and ${\bar{Res}}_{\rho }^{j}\left(t\right)$ are identified as:

(32) $$\begin{array}{c} {\underline{Res}}_{\rho }^{j}\left(t\right)=C^{\beta }\left(\left(\rho -1\right)+\overset{j}{\underset{k=1}{\sum }}\frac{c_{k}t^{\beta k}}{\beta^{k}k!}\right)-2t^{\beta }\left(\left(\rho -1\right)+\overset{j}{\underset{k=1}{\sum }}\frac{c_{k}t^{\beta k}}{\beta^{k}k!}\right)-\left(\rho -1\right)t^{\beta },\\ {\bar{Res}}_{\rho }^{j}\left(t\right)=C^{\beta }\left(\left(1-\rho \right)+\overset{j}{\underset{k=0}{\sum }}\frac{d_{k}t^{\beta k}}{\beta^{k}k!}\right)-2t^{\beta }\left(\left(1-\rho \right)+\overset{j}{\underset{k=0}{\sum }}\frac{d_{k}t^{\beta k}}{\beta^{k}k!}\right)-\left(1-\rho \right)t^{\beta }. \end{array}$$

For j=1, the first fractional residual functions ${\underline{Res}}_{\rho }^{1}\left(t\right)$ and ${\bar{Res}}_{\rho }^{1}\left(t\right)$ could be expressed as:

(33) $$\begin{array}{c} {\underline{Res}}_{\rho }^{1}\left(t\right)=C^{\beta }\left(\left(\rho -1\right)+\frac{c_{1}t^{\beta }}{\beta }\right)-\left(3t^{\beta }\left(\rho -1\right)+\frac{2c_{1}t^{2\beta }}{\beta }\right)=c_{1}-3t^{\beta }\left(\rho -1\right)-\frac{2c_{1}t^{2\beta }}{\beta },\\ {\bar{Res}}_{\rho }^{1}\left(t\right)=C^{\beta }\left(\left(1-\rho \right)+\frac{d_{1}t^{\beta }}{\beta }\right)-\left(3t^{\beta }\left(1-\rho \right)+\frac{2d_{1}t^{2\beta }}{\beta }\right)=d_{1}-3t^{\beta }\left(1-\rho \right)-\frac{2d_{1}t^{2\beta }}{\beta }. \end{array}$$

Solving the systems ${\underline{Res}}_{\rho }^{1}\left(0\right)=0$ and ${\bar{Res}}_{\rho }^{1}\left(0\right)=0$ gives $c_{1}=d_{1}=0$.

Again, to determine $c_{2}$ and $d_{2}$ set j=2 in (32), then taking into account the values of the obtained coefficients, yields

(34) $$\begin{array}{c} {\underline{Res}}_{\rho }^{2}\left(t\right)=C^{\beta }\left(\left(\rho -1\right)+\frac{c_{2}t^{2\beta }}{2\beta^{2}}\right)-\left(3t^{\beta }\left(\rho -1\right)+\frac{c_{2}t^{3\beta }}{\beta^{2}}\right)=\frac{c_{2}t^{\beta }}{\beta }-3t^{\beta }\left(\rho -1\right)-\frac{c_{2}t^{3\beta }}{\beta^{2}},\\ {\bar{Res}}_{\rho }^{2}\left(t\right)=C^{\beta }\left(\left(1-\rho \right)+\frac{d_{2}t^{2\beta }}{2\beta^{2}}\right)-\left(3t^{\beta }\left(1-\rho \right)+\frac{d_{2}t^{3\beta }}{\beta^{2}}\right)=\frac{d_{2}t^{\beta }}{\beta }-3t^{\beta }\left(1-\rho \right)-\frac{d_{2}t^{3\beta }}{\beta^{2}}. \end{array}$$

Applying the operator $C^{\beta }$ to both sides of (34) gives

(35) $$\begin{array}{c} C^{\beta }\left({\underline{Res}}_{\rho }^{2}\left(t\right)\right)=C^{\beta }\left(\frac{c_{2}t^{\beta }}{\beta }-3t^{\beta }\left(\rho -1\right)-\frac{c_{2}t^{3\beta }}{\beta^{2}}\right)=c_{2}-3\left(\rho -1\right)\beta -\frac{3c_{2}t^{2\beta }}{\beta },\\ C^{\beta }\left({\bar{Res}}_{\rho }^{2}\left(t\right)\right)=C^{\beta }\left(\frac{d_{2}t^{\beta }}{\beta }-3t^{\beta }\left(\rho -1\right)-\frac{d_{2}t^{3\beta }}{\beta^{2}}\right)=d_{2}-3\left(1-\rho \right)\beta -\frac{3d_{2}t^{2\beta }}{\beta }. \end{array}$$

According to $C^{\beta }{\underline{Res}}_{\rho }^{2}\left(0\right)=0$ and $C^{\beta }{\bar{Res}}_{\rho }^{2}\left(0\right)=0$, we have $c_{2}=3\left(\rho -1\right)\beta$ and $d_{2}=3\left(1-\rho \right)\beta$. By taking into account the obtained coefficients, for j=3, we have

(36) $$\begin{array}{c} C^{2\beta }\left({\underline{Res}}_{\rho }^{3}\left(t\right)\right)=C^{2\beta }\left(C^{\beta }\left(\left(\rho -1\right)+\frac{3\left(\rho -1\right)\beta t^{2\beta }}{2\beta^{2}}+\frac{c_{3}t^{3\beta }}{6\beta^{3}}\right)-\left(3t^{\beta }\left(\rho -1\right)+\frac{6\left(\rho -1\right)\beta t^{3\beta }}{2\beta^{2}}+\frac{2c_{3}t^{4\beta }}{6\beta^{3}}\right)\right)=c_{3}-18\left(\rho -1\right)\beta t^{\beta }-\frac{4c_{3}t^{2\beta }}{\beta },\\ C^{2\beta }\left({\bar{Res}}_{\rho }^{3}\left(t\right)\right)=C^{2\beta }\left(C^{\beta }\left(\left(1-\rho \right)+\frac{3\left(1-\rho \right)\beta t^{2\beta }}{2\beta^{2}}+\frac{d_{3}t^{3\beta }}{6\beta^{3}}\right)-\left(3t^{\beta }\left(1-\rho \right)+\frac{6\left(1-\rho \right)\beta t^{3\beta }}{2\beta^{2}}+\frac{2d_{3}t^{4\beta }}{6\beta^{3}}\right)\right)=d_{3}-18\left(1-\rho \right)\beta t^{\beta }-\frac{4d_{3}t^{2\beta }}{\beta }. \end{array}$$

Using the fact $C^{2\beta }{\underline{Res}}_{\rho }^{3}\left(0\right)=0$ and $C^{\beta }{\bar{Res}}_{\rho }^{3}\left(0\right)=0$ we have $c_{3}=0$ and $d_{3}=0$.

By the MATHEMATICA Software Package 12 and employing the process of Algorithm 1 for our present method, we deduced that
$c_{4}=18\left(\rho -1\right)\beta^{2}$,$d_{4}=18\left(1-\rho \right)\beta^{2}$,$c_{5}=0$,$d_{5}=0$,$c_{6}=180\left(\rho -1\right)\beta^{3}$,$d_{6}=180\left(1-\rho \right)\beta^{3}$,$c_{7}=0$,$d_{7}=0$,⋮⋮

Therefore, when j→∞ the fractional PS approximate solutions of (30) could be written as

(37) $$\begin{array}{c} {\underline{\omega }}_{\rho }\left(t\right)=\left(\rho -1\right)+\frac{3\left(\rho -1\right)t^{2\beta }}{2!\beta }+\frac{18\left(\rho -1\right)t^{4\beta }}{4!\beta^{2}}+\frac{180\left(\rho -1\right)t^{6\beta }}{6!\beta^{3}}+\cdots ,\\ {\bar{\omega }}_{\rho }\left(t\right)=\left(1-\rho \right)+\frac{3\left(1-\rho \right)t^{2\beta }}{2!\beta }+\frac{18\left(1-\rho \right)t^{4\beta }}{4!\beta^{2}}+\frac{180\left(1-\rho \right)t^{6\beta }}{6!\beta^{3}}+\cdots . \end{array}$$

In case β=1, the fractional expansions (37) reduced to the following classical expansions

(38) $$\begin{array}{c} {\underline{\omega }}_{\rho }\left(t\right)=\left(\rho -1\right)+\frac{3\left(\rho -1\right)t^{2}}{2}+\frac{3\left(\rho -1\right)t^{4}}{4\beta^{2}}+\frac{3\left(\rho -1\right)t^{6}}{12}+\cdots ,\\ {\bar{\omega }}_{\rho }\left(t\right)=\left(1-\rho \right)+\frac{3\left(1-\rho \right)t^{2}}{2}+\frac{3\left(1-\rho \right)t^{4}}{4}+\frac{3\left(1-\rho \right)t^{6}}{12}+\cdots . \end{array}$$

which converges to the exact solutions ${\underline{\omega }}_{\rho }\left(t\right)=\frac{1}{2}\left(\rho -1\right)\left(3e^{t^{2}}-1\right)$ and ${\bar{\omega }}_{\rho }\left(t\right)=\frac{1}{2}\left(1-\rho \right)\left(3e^{t^{2}}-1\right)$.

**Case II:** If $\omega \left(t\right)$ is $\left(2;\beta \right)$-fuzzy conformable differentiable, then the corresponding crisp system of fuzzy fractional IVPs (28) and (29) will be written in the following form

(39) $$\left\{\begin{array}{c} C^{\beta }{\underline{\omega }}_{\rho }\left(t\right)=2t^{\beta }{\bar{\omega }}_{\rho }\left(t\right)+\left(1-\rho \right)t^{\beta },\\ C^{\beta }{\bar{\omega }}_{\rho }\left(t\right)=2t^{\beta }{\underline{\omega }}_{\rho }\left(t\right)+\left(\rho -1\right)t^{\beta },\\ {\underline{\omega }}_{\rho }\left(0\right)=\rho -1,\;\;\;\;{\bar{\omega }}_{\rho }\left(0\right)=1-\rho . \end{array}\right.$$

The fuzzy exact solution at β=1 in the ρ-cut representation is ${\left[\omega \left(t\right)\right]}_{\rho }=\frac{1}{2}\left[1-\rho ,\rho -1\right]\left(1-3e^{-t^{2}}\right)$. By applying the RPS method, and using the j-th fractional residual functions ${\underline{Res}}_{\rho }^{j}\left(t\right)$ and ${\bar{Res}}_{\rho }^{j}\left(t\right)$ of the fractional IVPs, (39) could be expressed as

(40) $$\begin{array}{c} {\underline{Res}}_{\rho }^{j}\left(t\right)=C^{\beta }\left(\left(1-\rho \right)+\overset{j}{\underset{k=1}{\sum }}\frac{c_{k}t^{\beta k}}{\beta^{k}k!}\right)-3t^{\beta }\left(1-\rho \right)-\overset{j}{\underset{k=1}{\sum }}\frac{d_{k}t^{\beta \left(k+1\right)}}{\beta^{k}k!},\\ {\bar{Res}}_{\rho }^{j}\left(t\right)=C^{\beta }\left(\left(\rho -1\right)+\overset{j}{\underset{k=0}{\sum }}\frac{d_{k}t^{\beta k}}{\beta^{k}k!}\right)-3t^{\beta }\left(\rho -1\right)-\overset{j}{\underset{k=1}{\sum }}\frac{c_{k}t^{\beta \left(k+1\right)}}{\beta^{k}k!}. \end{array}$$

Following the same procedure as mentioned above, the first six coefficients $c_{k}$ and $d_{k}$, for k=1,2,3,4,5,6, are listed below. More coefficients can be computed in the same manner.
$c_{1}=0$,$d_{1}=0$,$c_{2}=3\beta \left(1-\rho \right)$,$d_{2}=3\beta \left(\rho -1\right)$,$c_{3}=0$,$d_{3}=0$,$c_{4}=18\left(\rho -1\right)\beta^{2}$,$d_{4}=18\left(1-\rho \right)\beta^{2}$,$c_{5}=0$,$d_{5}=0$,$c_{6}=180\left(1-\rho \right)\beta^{3}$,$d_{6}=180\left(\rho -1\right)\beta^{3}$,⋮⋮

Consequently, the sixth fractional PS approximate solutions for fractional IVPs system (39) have the forms

(41) $$\begin{array}{c} {\underline{\omega }}_{\rho }^{6}\left(t\right)=\left(\rho -1\right)+\frac{3\left(1-\rho \right)t^{2\beta }}{2!\beta }+\frac{18\left(\rho -1\right)t^{4\beta }}{4!\beta^{2}}+\frac{180\left(1-\rho \right)t^{6\beta }}{6!\beta^{3}},\\ {\bar{\omega }}_{\rho }^{6}\left(t\right)=\left(1-\rho \right)+\frac{3\left(\rho -1\right)t^{2\beta }}{2!\beta }+\frac{18\left(1-\rho \right)t^{4\beta }}{4!\beta^{2}}+\frac{180\left(\rho -1\right)t^{6\beta }}{6!\beta^{3}}. \end{array}$$

In particular, when β=1, the fractional expansions (37) reduce to following finite series expansions

(42) $$\begin{array}{c} {\underline{\omega }}_{\rho }^{6}\left(t\right)=\left(\rho -1\right)-\frac{3\left(\rho -1\right)t^{2}}{2}+\frac{3\left(\rho -1\right)t^{4}}{4}-\frac{\left(\rho -1\right)t^{6}}{4},\\ {\bar{\omega }}_{\rho }^{6}\left(t\right)=\left(1-\rho \right)-\frac{3\left(1-\rho \right)t^{2}}{2}+\frac{3\left(1-\rho \right)t^{4}}{4}-\frac{\left(1-\rho \right)t^{6}}{4}, \end{array}$$

which agree with the first six terms of the McLaurin series of the exact solutions, ${\underline{\omega }}_{\rho }\left(t\right)=\frac{1}{2}\left(1-\rho \right)\left(1-3e^{-t^{2}}\right)$ and ${\bar{\omega }}_{\rho }\left(t\right)=\frac{1}{2}\left(\rho -1\right)\left(1-3e^{-t^{2}}\right)$. Numerical simulation of the sixth fractional PS approximate solutions is performed for Application 2, case I at different values of β and ρ with some selected grid points with step size 0.2 on the interval $\left[0,1\right]$ as shown in Table 3.

For the purpose of numerical comparisons, the absolute errors were calculated for Application 2, case I using the RPS method with the reproducing kernel Hilbert space method (RKHSM) method [53], for fixed value of ρ, and different values of t, where $t\in \left\{0,0.2,0.4,0.6,0.8,1\right\}$ as shown in Table 4.

It’s clear that from this table our method in comparison with the mentioned method is much better with a view to accuracy and applicability.

**Figure 2:**
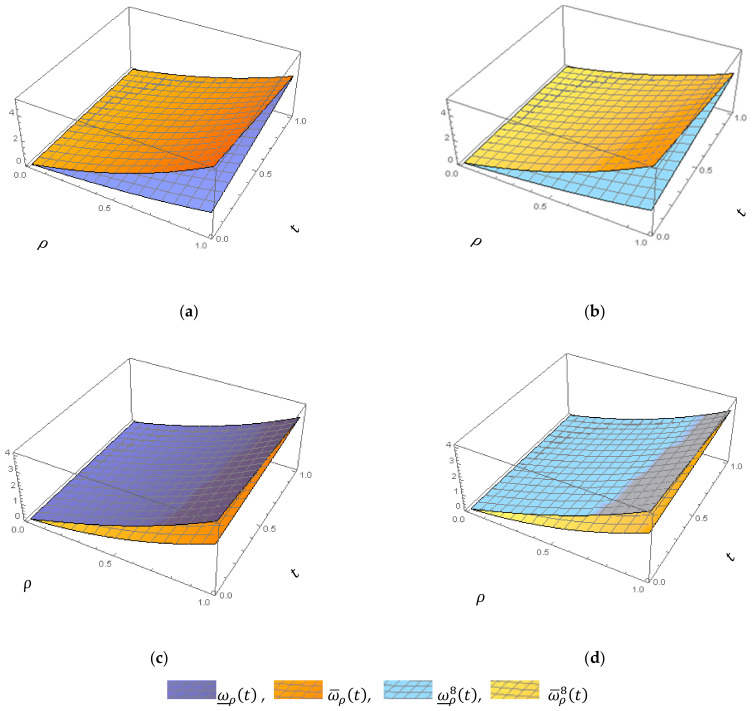
(**a**) 3D-Surfaces Plot of $\left[{\underline{\omega }}_{\rho }\left(t\right),{\bar{\omega }}_{\rho }\left(t\right)\right]$ at β=1, case I. (**b**) 3D-Surfaces Plot of $\left[{\underline{\omega }}_{\rho }^{8}\left(t\right),{\bar{\omega }}_{\rho }^{8}\left(t\right)\right]$ at β=1, case I. (**c**) 3D-Surfaces Plot of $\left[{\underline{\omega }}_{\rho }\left(t\right),{\bar{\omega }}_{\rho }\left(t\right)\right]$ at β=1, case II. (**d**) 3D-Surfaces Plot of $\left[{\underline{\omega }}_{\rho }^{8}\left(t\right),{\bar{\omega }}_{\rho }^{8}\left(t\right)\right]$ at β=1, case II; for Application 1.

**Figure 3:**
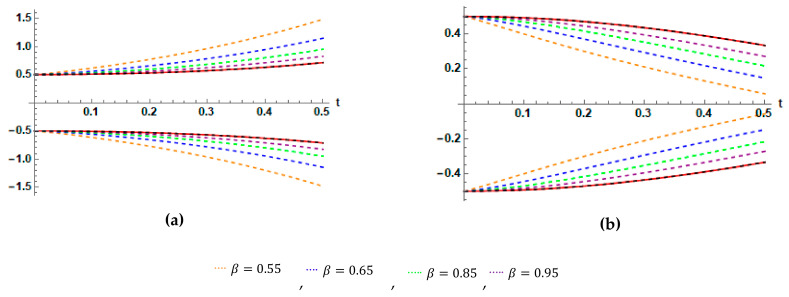
(**a**) Plots of 0.5-cut representations of the fuzzy exact solution $\left[{\underline{\omega }}_{0.5}\left(t\right),{\bar{\omega }}_{0.5}\left(t\right)\right]$ and the fuzzy approximate solution $\left[{\underline{\omega }}_{0.5}^{6}\left(t\right),{\bar{\omega }}_{0.5}^{6}\left(t\right)\right]$, case I. (**b**) Plots of 0.5-cut representations of the fuzzy exact solution $\left[{\underline{\omega }}_{0.5}\left(t\right),{\bar{\omega }}_{0.5}\left(t\right)\right]$ and the fuzzy approximate solution $\left[{\underline{\omega }}_{0.5}^{6}\left(t\right),{\bar{\omega }}_{0.5}^{6}\left(t\right)\right]$, case II, for Application 2, at different values of β.

**Figure 4:**
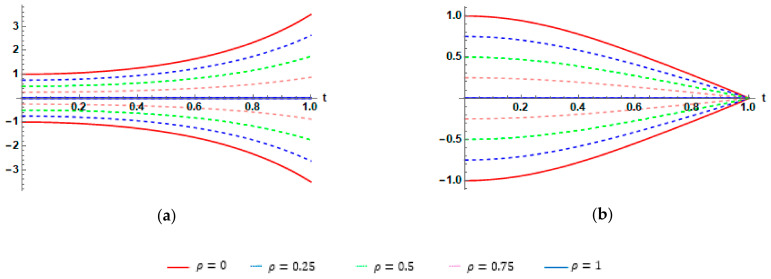
(**a**) Plots of ρ-cut representations of the fuzzy exact solution $\left[{\underline{\omega }}_{\rho }\left(t\right),{\bar{\omega }}_{\rho }\left(t\right)\right]$ and the fuzzy approximate solution $\left[{\underline{\omega }}_{\rho }^{8}\left(t\right),{\bar{\omega }}_{\rho }^{8}\left(t\right)\right]$, case I. (**b**) Plots of ρ-cut representations of the fuzzy exact solution $\left[{\underline{\omega }}_{\rho }\left(t\right),{\bar{\omega }}_{\rho }\left(t\right)\right]$ and fuzzy approximate solution $\left[{\underline{\omega }}_{\rho }^{8}\left(t\right),{\bar{\omega }}_{\rho }^{8}\left(t\right)\right]$, case II, for Application 2, at β=1..

Graphically, to demonstrate the impact of parameters β and ρ on the behavior solutions, we plot the fuzzy exact and fuzzy sixth approximate solutions for Application 2, as shown in Figure 2, Figure 3 and Figure 4

## 6. Conclusions

In this analysis, fuzzy approximate solutions were created and studied for a certain class of FFDEs with fuzzy initial data by means of RPS approach under fuzzy conformable differentiability. The methodology for solving the target problem was based on converting it into two crisp systems of ordinary IVPs. Using the proposed approach, the fractional PS solutions were given in the parametric forms for fuzzy fractional IVPs. The benefit of employing the present approach is that it provides a rapidly convergent fractional PS with easily computable components using symbolic computation software without avoiding round-off errors and sometimes could be expressed in closed form. Two different fuzzy initial data are solved to show the applicability of the proposed approach and to test the accuracy of the RPS approach. The obtained results are compared with other existing approaches. Simulations of the obtained results are discussed quantitatively and graphically and shown that the behavior of the approximate solutions for different values of β and ρ continuously tends to the exact solutions. Therefore, the RPS approach is straightforward without using mathematical conditions in obtaining solutions of conformable FFDEs.

## Figures and Tables

**Table 1:** Absolute errors for Application 1, case I.

$t_{i}$	$E_{8}\left({\underline{\omega }}_{\rho }\right)=\left|{\underline{\omega }}_{\rho }\left(t\right)-{\underline{\omega }}_{\rho }^{8}\left(t\right)\right|\;$		
	ρ=0	ρ=0.5	ρ=1
0.16	0.0	0.0	0.0
0.32	$1.00\times 10^{-10}$	0.0	$3.0\times 10^{-10}$
0.48	$4.000\times 10^{-9}$	$6.000\times 10^{-9}$	$8.00\times 10^{-9}$
0.64	$5.300\times 10^{-8}$	$7.900\times 10^{-8}$	$1.06\times 10^{-7}$
0.80	$4.020\times 10^{-7}$	$6.030\times 10^{-7}$	$8.04\times 10^{-7}$
0.96	$2.109\times 10^{-6}$	$3.164\times 10^{-6}$	$4.21\times 10^{-6}$
$t_{i}$	$E_{8}\left({\bar{\omega }}_{\rho }\right)=\left|{\bar{\omega }}_{\rho }\left(t\right)-{\bar{\omega }}_{\rho }^{8}\left(t\right)\right|$		
	ρ=0	ρ=0.5	ρ=1
0.16	0.0	0.0	0.0
0.32	$4.00\times 10^{-10}$	0.0	$3.00\times 10^{-10}$
0.48	$1.20\times 10^{-8}$	$1.00\times 10^{-8}$	$8.00\times 10^{-9}$
0.64	$1.59\times 10^{-7}$	$1.33\times 10^{-7}$	$1.06\times 10^{-7}$
0.80	$1.20\times 10^{-6}$	$1.00\times 10^{-6}$	$8.04\times 10^{-7}$
0.96	$6.32\times 10^{-6}$	$5.27\times 10^{-6}$	$4.21\times 10^{-6}$

**Table 2:** Absolute errors for Application 1, case II.

$t_{i}\;$	$E_{8}\left({\underline{\omega }}_{\rho }\right)=\left|{\underline{\omega }}_{\rho }\left(t\right)-{\underline{\omega }}_{\rho }^{8}\left(t\right)\right|\;$		
	ρ=0	ρ=0.5	ρ=1
0.16	0.0	0.0	0.0
0.32	$4.00\times 10^{-10}$	0.0	0.0
0.48	$1.10\times 10^{-8}$	$1.00\times 10^{-8}$	$8.00\times 10^{-9}$
0.64	$1.53\times 10^{-7}$	$1.29\times 10^{-7}$	$1.06\times 10^{-7}$
0.80	$1.15\times 10^{-7}$	$9.75\times 10^{-7}$	$8.04\times 10^{-7}$
0.96	$5.96\times 10^{-6}$	$5.09\times 10^{-6}$	$4.22\times 10^{-6}$
$t_{i}$	$E_{8}\left({\bar{\omega }}_{\rho }\right)=\left|{\bar{\omega }}_{\rho }\left(t\right)-{\bar{\omega }}_{\rho }^{8}\left(t\right)\right|$		
	ρ=0	ρ=0.5	ρ=1
0.16	0.0	0.0	0.0
0.32	0.0	0.0	0.0
0.48	$4.00\times 10^{-9}$	$6.00\times 10^{-9}$	$8.00\times 10^{-9}$
0.64	$5.90\times 10^{-8}$	$8.30\times 10^{-8}$	$1.06\times 10^{-7}$
0.80	$4.61\times 10^{-7}$	$6.32\times 10^{-7}$	$8.04\times 10^{-7}$
0.96	$2.48\times 10^{-6}$	$3.35\times 10^{-6}$	$4.22\times 10^{-6}$

**Table 3:** Numerical results of $\left[{\underline{\omega }}_{\rho }^{6}\left(t\right),{\bar{\omega }}_{\rho }^{6}\left(t\right)\right]$ for Application 2, case II.

$t_{i}$	$\left[{\underline{\omega }}_{0.5}^{6}\left(t\right),{\bar{\omega }}_{0.5}^{6}\left(t\right)\right]$			
	β=1	β=0.95	β=0.85	β=0.75
0.2	−0.470592000	−0.463808920	−0.444926104	−0.415678602
0.4	−0.389088000	−0.373553570	−0.335280912	−0.284887162
0.6	−0.272767991	−0.252608283	−0.206098948	−0.149497486
0.8	−0.140831991	−0.120431908	−0.074024317	−0.017241246
0.2	0.470592000	0.463808920	0.444926104	0.415678602
0.4	0.389088000	0.373553570	0.335280912	0.284887162
0.6	0.272767991	0.252608283	0.206098948	0.149497486
0.8	0.140831991	0.120431908	0.074024317	0.017241246
$t_{i}$	$\left[{\underline{\omega }}_{0.75}^{6}\left(t\right),{\bar{\omega }}_{0.75}^{6}\left(t\right)\right]$			
	β=1	β=0.95	β=0.85	β=0.75
0.2	−0.235296	−0.231904460	−0.222463052	−0.207839301
0.4	−0.194544	−0.186776785	−0.167640456	−0.142443581
0.6	−0.136384	−0.126304141	−0.103049474	−0.074748743
0.8	−0.070416	−0.060215954	−0.037012158	−0.008620623
0.2	0.235296	0.231904460	0.222463052	0.207839301
0.4	0.194544	0.186776785	0.167640456	0.142443581
0.6	0.136384	0.126304141	0.103049474	0.074748743
0.8	0.070416	0.060215954	0.037012158	0.008620623

**Table 4:** Numerical comparison of absolute errors of Application 2 case II.

	$t_{i}$	ρ=0.25		ρ=0.75	
		RPSM	RKHSM	RPSM	RKHSM
${\underline{\omega }}_{\rho }\left(t\right)$	0	0	0	0	0
	0.2	$3.63043\times 10^{-14}$	$7.02959\times 10^{-6}$	$1.21292\times 10^{-14}$	$2.34320\times 10^{-6}$
	0.4	$5.87412\times 10^{-10}$	$7.48175\times 10^{-6}$	$1.95804\times 10^{-10}$	$2.49392\times 10^{-6}$
	0.6	$1.67352\times 10^{-7}$	$8.64832\times 10^{-6}$	$5.57842\times 10^{-8}$	$2.88277\times 10^{-6}$
	0.8	$9.08417\times 10^{-6}$	$1.10884\times 10^{-5}$	$3.02806\times 10^{-6}$	$3.69612\times 10^{-6}$
	1	$1.98129\times 10^{-5}$	$1.57693\times 10^{-5}$	$6.60429\times 10^{-6}$	$5.25645\times 10^{-6}$
	$t_{i}$	ρ=0.25		ρ=0.75	
		RPSM	RKHSM	RPSM	RKHSM
${\bar{\omega }}_{\rho }\left(t\right)$	0	0	0	0	0
	0.2	$3.63043\times 10^{-14}$	$7.02959\times 10^{-6}$	$1.21292\times 10^{-14}$	$2.34320\times 10^{-6}$
	0.4	$5.87412\times 10^{-10}$	$7.48175\times 10^{-6}$	$1.95804\times 10^{-10}$	$2.49392\times 10^{-6}$
	0.6	$1.67352\times 10^{-7}$	$8.64832\times 10^{-6}$	$5.57842\times 10^{-8}$	$2.88277\times 10^{-6}$
	0.8	$9.08417\times 10^{-6}$	$1.10884\times 10^{-5}$	$3.02806\times 10^{-6}$	$3.69612\times 10^{-6}$
	1	$1.98129\times 10^{-5}$	$1.57693\times 10^{-5}$	$6.68949\times 10^{-6}$	$5.25645\times 10^{-6}$

## Data Availability

Not applicable.

## References

[1] Zadeh L.A. (1965). Fuzzy sets. Inf. Control.

[2] Kandel A. (1986). Fuzzy Mathematical Techniques with Applications.

[3] Mendel J.M. (1995). Fuzzy logic systems for engineering: A tutorial. Proc. IEEE.

[4] Bede B., Gal S.G. (2005). Generalizations of the differentiability of fuzzy number value functions with applications to fuzzy differential equations. Fuzzy Sets Syst..

[5] Chalco-Cano Y., Román-Flores H. (2008). On new solutions of fuzzy differential equations. Chaos Solitons Fractals.

[6] Kaleva O. (1987). Fuzzy differential equations. Fuzzy Sets Syst..

[7] Hasan S., Al-Smadi M., El-Ajou A., Momani S., Hadid S., Al-Zhour Z. (2021). Numerical approach in the Hilbert space to solve a fuzzy Atangana-Baleanu fractional hybrid system. Chaos Solitons Fractals.

[8] Al-Smadi M., Abu Arqub O., Zeidan D. (2021). Fuzzy fractional differential equations under the Mittag-Leffler kernel differential operator of the ABC approach: Theorems and applications. Chaos Solitons Fractals.

[9] Al-Smadi M., Abu Arqub O., Gaith M. (2021). Numerical simulation of telegraph and Cattaneo fractional-type models using adaptive reproducing kernel framework. Math. Methods Appl. Sci..

[10] Caputo M. (1967). Linear models of dissipation whose Q is almost frequency independent: Part II. Geophys. J. Inter..

[11] Abu Arqub O., Al-Smadi M. (2020). An adaptive numerical approach for the solutions of fractional advection-diffusion and dispersion equations in singular case under Riesz’s derivative operator. Phys. A Stat. Mech. Its Appl..

[12] Al-Smadi M., Abu Arqub O. (2019). Computational algorithm for solving fredholm time-fractional partial integrodifferential equations of dirichlet functions type with error estimates. Appl. Math. Comput..

[13] Akinyemi L., Iyiola O.S., Akpan U. (2020). Iterative methods for solving fourth- and sixth-order time-fractional Cahn-Hillard equation. Math. Methods Appl. Sci..

[14] Akinyemi L., Iyiola O.S. (2020). Exact and approximate solutions of time-fractional models arising from physics via Shehu transform. Math. Methods Appl. Sci..

[15] Akinyemi L., Iyiola O.S. (2020). A reliable technique to study nonlinear time-fractional coupled Korteweg–de Vries equations. Adv. Differ. Equ..

[16] Şenol M., Iyiola O.S., Daei Kasmaei H., Akinyemi L. (2019). Efficient analytical techniques for solving time-fractional nonlinear coupled Jaulent–Miodek system with energy-dependent Schrödinger potential. Adv. Differ. Equ..

[17] Akinyemi L., Iyiola O.S. (2021). Analytical Study of (3+1)-Dimensional Fractional-Reaction Diffusion Trimolecular Models. Int. J. Appl. Comput. Math..

[18] Hasan S., El-Ajou A., Hadid S., Al-Smadi M., Momani S. (2020). Atangana-Baleanu fractional framework of reproducing kernel technique in solving fractional population dynamics system. Chaos Solitons Fractals.

[19] Agarwal R.P., Lakshmikantham V., Nieto J.J. (2010). On the concept of solution for fractional differential equations with uncertainty. Nonlinear Anal. Theory Methods Appl..

[20] Al-Smadi M., Freihat A., Abu Arqub O., Shawagfeh N. (2015). A novel multistep generalized differential transform method for solving fractional-order Lü chaotic and hyperchaotic systems. J. Comput. Anal. Appl..

[21] Altawallbeh Z., Al-Smadi M., Komashynska I., Ateiwi A. (2018). Numerical Solutions of Fractional Systems of Two-Point BVPs by Using the Iterative Reproducing Kernel Algorithm. Ukr. Math. J..

[22] Khodadadi E., Çelik E. (2013). The variational iteration method for fuzzy fractional differential equations with uncertainty. Fixed Point Theory Appl..

[23] Momani S., Djeddi N., Al-Smadi M., Al-Omari S. (2021). Numerical investigation for Caputo-Fabrizio fractional Riccati and Bernoulli equations using iterative reproducing kernel method. Appl. Numer. Math..

[24] Saadeh R., Alaroud M., Al-Smadi M., Ahmad R.R., Salma Din U.K. (2019). Application of fractional residual power series algorithm to solve Newell–Whitehead–Segel equation of fractional order. Symmetry.

[25] Al-Smadi M. (2021). Fractional residual series for conformable time-fractional Sawada–Kotera–Ito, Lax, and Kaup–Kupershmidt equations of seventh order. Math. Methods Appl. Sci..

[26] Freihet A., Hasan S., Alaroud M., Al-Smadi M., Ahmad R.R., Salma Din U.K. (2019). Toward computational algorithm for time-fractional Fokker–Planck models. Adv. Mech. Eng..

[27] Al-Smadi M., Abu Arqub O., Hadid S. (2020). Approximate solutions of nonlinear fractional Kundu-Eckhaus and coupled fractional massive Thirring equations emerging in quantum field theory using conformable residual power series method. Phys. Scr..

[28] Al-Smadi M., Abu Arqub O., Momani S. (2020). Numerical computations of coupled fractional resonant Schrödinger equations arising in quantum mechanics under conformable fractional derivative sense. Phys. Scr..

[29] Kumar A., Kumar S., Singh M. (2016). Residual power series method for fractional Sharma-Tasso-Olever equation. Commun. Numer. An..

[30] Al-Smadi M., Djeddi N., Momani S., Al-Omari S., Araci S. (2021). An attractive numerical algorithm for solving nonlinear Caputo–Fabrizio fractional Abel differential equation in a Hilbert space. Adv. Differ. Equ..

[31] Agarwal P., Jain S., Ahmad B., Al-Omari S. (2015). Certain recent fractional integral inequalities associated with the hypergeometric operators. J. King Saud Uni. Sci..

[32] Al-Smadi M., Abu Arqub O., Shawagfeh N., Momani S. (2016). Numerical investigations for systems of second-order periodic boundary value problems using reproducing kernel method. Appl. Math. Comput..

[33] Hasan S., Djeddi N., Al-Smadi M., Al-Omari S., Momani S., Fulga A. (2021). Numerical solvability of generalized Bagley–Torvik fractional models under Caputo–Fabrizio derivative. Adv. Differ. Equ..

[34] Hasan S., Al-Smadi M., Freihet A., Momani S. (2019). Two computational approaches for solving a fractional obstacle system in Hilbert space. Adv. Differ. Equ..

[35] Al-Smadi M. (2018). Simplified iterative reproducing kernel method for handling time-fractional BVPs with error estimation. Ain Shams Eng. J..

[36] Khalil R., Al Horani M., Yousef A., Sababheh M. (2014). A new definition of fractional derivative. J. Comput. Appl. Math..

[37] Alaroud M., Al-Smadi M., Ahmad R.R., Salma Din U.K. (2019). An analytical numerical method for solving fuzzy fractional Volterra integro-differential equations. Symmetry.

[38] Alaroud M., Al-Smadi M., Ahmad R.R., Salma Din U.K. (2018). Computational optimization of residual power series algorithm for certain classes of fuzzy fractional differential equations. Int. J. Differ. Equ..

[39] Alaroud M. (2021). Application of Laplace residual power series method for approximate solutions of fractional IVP’s. Alex. Eng. J..

[40] Clemence-Mkhope D.P., Clemence-Mkhope B.G.B. (2021). The Limited Validity of the Conformable Euler Finite Difference Method and an Alternate Definition of the Conformable Fractional Derivative to Justify Modification of the Method. Math. Comput. Appl..

[41] Momani S., Abu Arqub O., Freihat A., Al-Smadi M. (2016). Analytical approximations for Fokker-Planck equations of fractional order in multistep schemes. Appl. Comput. Math..

[42] Freihet A., Hasan S., Al-Smadi M., Gaith M., Momani S. (2019). Construction of fractional power series solutions to fractional stiff system using residual functions algorithm. Adv. Differ. Equ..

[43] Al-Smadi M., Abu Arqub O., Hadid S. (2020). An attractive analytical technique for coupled system of fractional partial differential equations in shallow water waves with conformable derivative. Commun. Theor. Phys..

[44] Moaddy K., Al-Smadi M., Hashim I. (2015). A novel representation of the exact solution for differential algebraic equations system using residual power-series method. Discret. Dyn. Nat. Soc..

[45] Komashynska I., Al-Smadi M., Abu Arqub O., Momani S. (2016). An efficient analytical method for solving singular initial value problems of nonlinear systems. Appl. Math. Inf. Sci..

[46] Harir A., Melliani S., Chadli L.S. (2020). Fuzzy generalized conformable fractional derivative. Adv. Fuzzy Syst..

[47] Al-Smadi M., Freihat A., Khalil H., Momani S., Khan R.A. (2017). Numerical Multistep Approach for Solving Fractional Partial Differential Equations. Int. J. Comput. Methods.

[48] Gumah G., Naser M., Al-Smadi M., Al-Omari S., Baleanu D. (2020). Numerical solutions of hybrid fuzzy differential equations in a Hilbert space. Appl. Numer. Math..

[49] Gumah G., Naser M., Al-Smadi M., Al-Omari S. (2018). Application of reproducing kernel Hilbert space, method for solving second-order fuzzy Volterra integro-differential equation. Adv. Differ. Equ..

[50] Chandak S., Suthar D.L., Al-Omari S.K., Gulyaz-Ozyurt S. (2021). Estimates of classes of generalized special functions and their application in the fractional (k,s)-calculus theory. J. Funct. Spaces Vol..

[51] Alaroud M., Tahat N., Al-Omari S., Suthar D.L., Gulyaz-Ozyurt S. (2021). An attractive approach associated with transform functions for solving certain fractional Swift-Hohenberg equation. J. Funct. Spaces..

[52] Al-Omari S., Dayalal Suthar D., Araci S. (2021). A fractional *q*-integral operator associated with a certain class of *q*-Bessel functions and *q*-generating series. Adv. Differ. Equ..

[53] Al-Smadi M., Dutta H., Hasan S., Momani S. (2021). On numerical approximation of Atangana-Baleanu-Caputo fractional integro-differential equations under uncertainty in Hilbert Space. Math. Model. Nat. Phenom..

